# Intermetallic Platinum‐Calcium Alloy Breaks the Activity‐Stability Trade‐Off in Fuel Cell for Enhanced Performance

**DOI:** 10.1002/smll.202503692

**Published:** 2025-06-05

**Authors:** Caleb Gyan‐Barimah, Kapil Dhaka, Ha‐Young Lee, Yi Wei, Muhammad Irfansyah Maulana, Jeong‐Hoon Yu, Bo Yu, Kai S. Exner, Jong‐Sung Yu

**Affiliations:** ^1^ Department of Energy Science & Engineering Daegu Gyeongbuk Institute of Science and Technology (DGIST) Daegu 42988 Republic of Korea; ^2^ Faculty of Chemistry, Theoretical Catalysis and Electrochemistry University Duisburg – Essence 451141 Essen Germany; ^3^ UE Science R7‐507, 333 Techno Jungang‐Daero Daegu 42988 Republic of Korea; ^4^ Cluster of Excellence RESOLV 44801 Bochum Germany; ^5^ Center for Nanointegration (CENIDE) Duisburg‐Essen 47057 Duisburg Germany

**Keywords:** activity, electrochemistry, fuel Cells, intermetallic alloys, stability

## Abstract

The realization of proton exchange membrane fuel cell (PEMFC) as a replacement for combustion engines and batteries in transportation applications demands a catalyst that is not only active but also highly stable throughout the vehicle's longevity. Alloys of platinum with alkaline earth metals have been identified to be active and highly stable owing to their high vacancy formation energies, but their synthesis in nanoparticle form has proven challenging, which discourages most researchers from exploring this area. In this work, the synthesis, characterization, and PEMFC test of platinum‐calcium (PtCa) nanoparticles prepared through the solution phase technique are reported. The PtCa catalyst reported here exhibits an intermetallic ordered atomic arrangement with a core–shell configuration, resulting in a specific rated power of 9 W mg_Pt_^−1^ at 0.67 V based on the cathode loading under H_2_‐air conditions. The reported catalyst also surpasses the US Department of Energy (DOE) 2025 mass activity target with an 81% retention in practical fuel cells after 30 000 durability cycles. This catalyst holds great potential to substitute the Pt‐transition alloy catalysts which have thus far fallen short of meeting commercial standards.

## Graphical abstract

A solution‐phase synthesized platinum‐alkaline earth metal alloy (PtCa) has been designed as an alternative to platinum‐transition metal alloys. The resulting alloy exhibits a structurally ordered arrangement which simultaneously achieves high activity and stability.

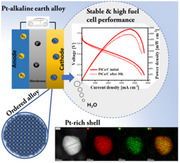

## 1 Introduction

The Energy Information Administration's data shows that the transportation of people and goods accounts for ≈25% of all energy consumption in the world.^[^ ^1^ ^]^ However, most of the fuel employed in this industry is based on petroleum derivatives which produce greenhouse gases as bi‐products.^[^ ^2^, ^3^ ^]^ To make this industry environmentally friendly, clean energy systems such as fuel cells must be employed.^[^ ^4^, ^5^, ^6^ ^]^ The fuel cell relies mainly on the use of platinum (Pt) nanoparticles (NPs) on carbon support as the anode and cathode catalysts.^[^ ^3^, ^5^, ^6^, ^7^, ^8^, ^9^, ^10^, ^11^, ^12^, ^13^, ^14^ ^]^ However, if the entire annual global production of Pt was focused on the production of fuel cell vehicles, fewer than 10 million vehicles would be produced each year.^[^ ^15^ ^]^ In the quest to mitigate problems associated with Pt, researchers have resorted to various strategies such as developing non‐Pt‐based catalysts as well as reducing the amount of Pt through alloying with transition metals.^[^ ^16^, ^17^ ^]^ However, the durability of these alloys remains a challenge.^[^ ^2^, ^18^ ^]^

To obtain a highly stable catalyst, one should consider the alloying energy (A_e_) and the dissolution potential. Data from the Materials Project database shows that alloys of Pt with rare earth metals (Y, La, Gd), and alkaline earth metals (Ca, Mg, Sr) can be great alternatives to the late transition metals.^[^ ^19^, ^20^, ^21^, ^22^, ^23^, ^24^, ^25^ ^]^ This is because alkaline earth metals for instance are extremely oxyphilic and much more electropositive compared to the late 3d transition metals. Hence, a high negative alloying energy (−A_e_) is required for their formation.^[^ ^26^, ^27^ ^]^ Alkaline earth metals (Mg, Ca) compared to Pt are very cheap, light in weight, and relatively abundant, making them good candidates to reduce the amount of Pt utilized. In addition, the high electronegativity difference between Pt and alkaline earth metals as well as the size difference introduces some ligand and strain effects between the underlayer alkaline earth metal atoms and the surface Pt atoms which significantly boosts the ORR activity. However, it is extremely difficult to synthesize clean Pt‐alkaline earth metals because of their high negative reduction potential. As proposed by Cui et al.,^[^ ^27^ ^]^ the reduction of their appropriate precursors requires either the use of an ultra‐fast reducing agent such as organoborohydrides or thermal annealing of the metal precursors at high temperatures above 700 °C. Tetteh et al. reported for the first time a Pt─Mg alloy with high stability.^[^ ^28^ ^]^ They achieved this alloy system by heating metallic Mg powder with Pt precursors at 650 °C. However, considering the high surface energy of Pt, alloying Pt with other alkaline earth metals such as Ca requires heating at temperatures above 1000 °C. Furthermore, work done by Vej‐Hansen and Greeley et al. through experimental and theoretical calculations shows that Pt_5_Ca and Pt_5_Sr are highly active for ORR.^[^ ^19^, ^29^, ^30^, ^31^ ^]^ The synthesis of particulate Pt─Ca alloy should be performed through simple steps with the optimum combination of special conditions such as a suitable aprotic non‐aqueous solvent, reducing temperature, and reducing agent. Also, Ca has a very high negative reduction potential (−2.87 V vs SHE) compared to Mg and thus is very difficult to reduce at room temperature employing common reducing agents like glucose, ascorbic acid, and phloroglucinol among others. Kanady et. al.^[^ ^31^ ^]^ employed a KCl matrix for the protection of Pt‐alkali metal, specifically Pt_2_Na, Pt‐rare‐earth metal alloys (Pt_3_Y, Pt_3_Lu), and Au_2_Y at high temperatures. However, particle‐to‐particle agglomeration can occur during the washing‐off of the KCl matrix. Other groups have resorted to the use of extended surfaces of Pt‐alkaline earth metals such as commercial Pt_5_Ca, but in such cases, there is a poor dispersion associated with these surfaces and thus, it would likely not be employed in practical fuel cells.^[^ ^30^, ^32^, ^33^ ^]^

Herein, we have synthesized highly ordered PtCa nanoparticles (NPs) encapsulated in a Pt‐rich shell through the solution phase different from the commercial Pt_5_Ca disks.^[^ ^19^ ^]^ Our PtCa/C catalyst exhibits a half cell mass activity of 0.80 A mg^−1^_Pt_ at 0.9 V which is 3 times higher than commercial Pt/C (0.31 A mg^−1^_Pt_). The PtCa/C, when employed as a cathode catalyst in a practical membrane electrode assembly (MEA) single cell, exhibits a high power density of 1755 mW cm^−2^ with only a 10% loss in peak power performance after 30 000 cycles with reference to DOE protocols.^[^ ^34^ ^]^ Theoretical calculations show that the introduction of Ca affects the relative stability of the reaction intermediates, which propels a boost in both the activity and stability of Pt.

## 2 Results and Discussion

### 2.1 Materials, Synthesis and Characterization

**Figure 1:**
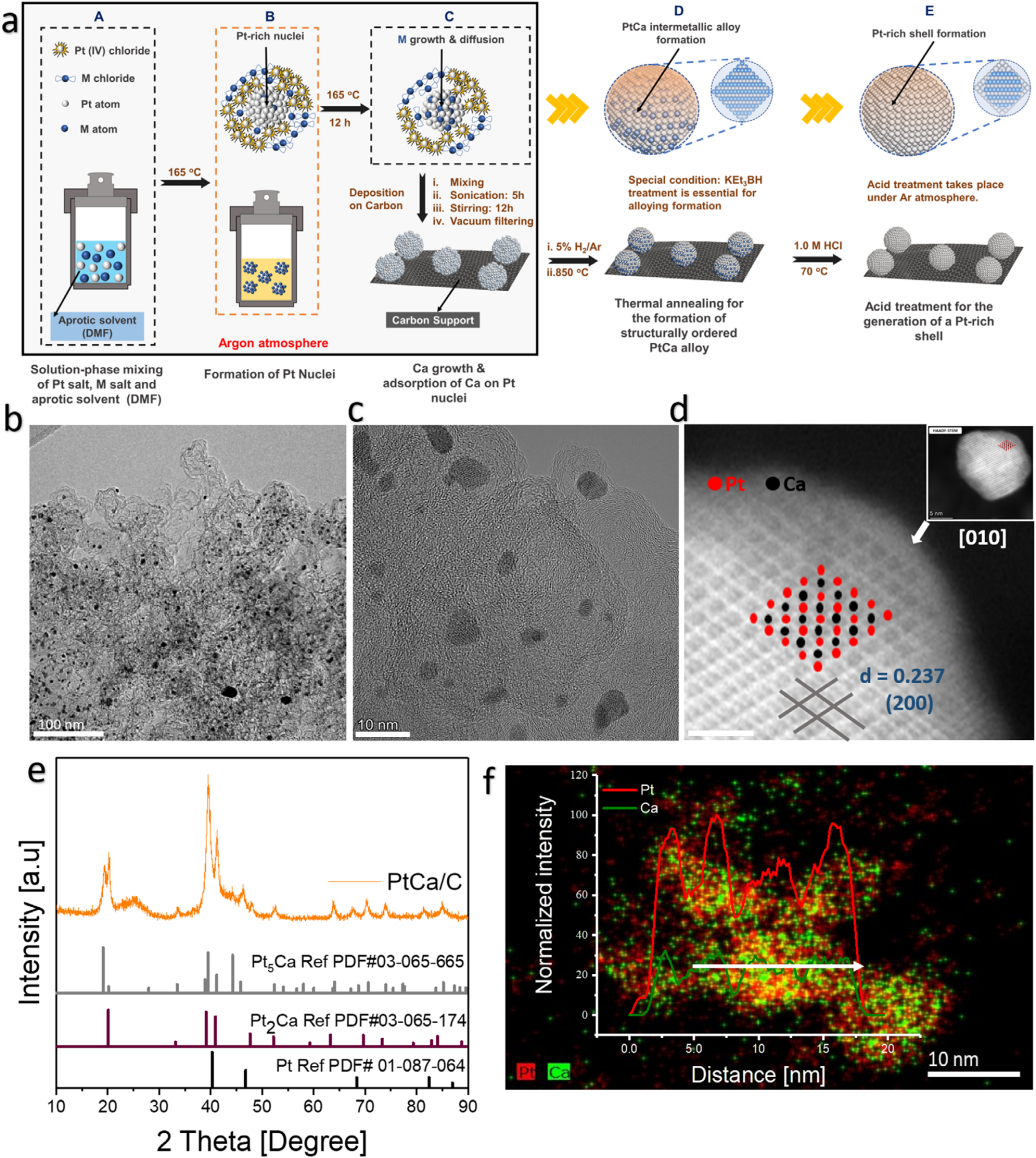
Structural characterization a) Schematic diagram showing the synthesis of PtCa NPs. TEM images of b) PtCa/C NPs, c) HR‐TEM image and d) HAADF‐STEM image showing the orderly arrangement of the Pt (bright spots) and Ca (dark spots) atoms, e) XRD patterns and f) STEM‐EDS line scan of the PtCa/C synthesized after 850 °C annealing treatment.

The PtCa/C alloy is synthesized via a carefully designed five‐step process (**Figure** **1**a), beginning with step A‐C, a solvothermal approach under air‐free conditions, followed by high‐temperature annealing (850–900 °C) in 5% H_2_ atmosphere (Step D) and the creation of a Pt‐rich skin (Step E). Pt(IV)chloride and calcium chloride (CaCl_2_) serve as precursors, DMF as the solvent, and NaBH_4_ as the reducing agent. In step A, all the precursors are weighed out as specified in the supporting information, sonicated for 30 min, and transferred to a Teflon‐lined autoclave. In the early stages of heating (Step B), the mixture is heated at 165 °C, which is above the boiling point of the solvent (153 °C). At this point, Pt nuclei begin to form first as a result of the decomposition of the Pt(IV)chloride. In step C, the formation of Pt nuclei is accompanied by the diffusion and adsorption of Ca nuclei surrounded by the DMF molecules. It is important to highlight that steps A‐C occur under Ar atmosphere to prevent oxygen influence.

In step D, the as‐prepared material is then annealed at a high temperature (850 – 900 °C) on a high surface area carbon support (Ketjen Black EC600J) to reduce the degree of aggregation. If Vulcan carbon (VC) is employed as support, severe aggregation can be observed with particle sizes ranging from 50 to 200 nm (Figure S1, Supporting Information). The annealing process is followed by acid etching in 1.0 M HCl at 70 °C (step E) to remove excess and unreacted CaCl_2_, calcium (Ca), and calcium oxide (CaO) formed as well as to create a Pt overlayer (Figure S2, Supporting Information). To test the flexibility of our method, we also synthesize other Pt‐alkaline earth (PtSr and PtBa) and Pt‐rare earth (Pt_3_Y) alloys (Figures S3–S5, Supporting Information) simply by maintaining all other reaction conditions and replacing the CaCl_2_ with either, SrCl_2_, BaCl_2_ or YCl_3_.

The morphology and structure of the as‐synthesized NPs are characterized by transmission electron microscopy (TEM), X‐ray absorption spectroscopy (XAS), scanning transmission electron microscopy‐energy dispersive X‐ray spectroscopy (STEM‐EDS) line scan, and mapping. Figure 1b shows the TEM image of the PtCa/C. The NPs are evenly distributed on the carbon support with an average particle size of 6.15 nm and varying particle sizes (Figure S6, Supporting Information). The varying particle sizes suggest that our prepared catalyst exists as a mixed phase. Due to this, we simply refer to it as PtCa/C. TEM and High‐resolution TEM (HR‐TEM) images as well as the high‐angle annular dark‐field (HAADF‐STEM) image (Figure 1c,d; Figure S7, Supporting Information) reveal the high crystallinity and ordered arrangement of the atoms in the alloy, as well as the incorporation of Ca into the Pt lattice, evidenced by the Z‐contrast in the HAADF‐STEM image, where the Pt atoms are in bright contrast to the dark Ca atoms (Figure 1d; Figure S7, Supporting Information). Here, the successful incorporation of Ca atoms into the Pt lattice is expected to introduce some strain onto the surface Pt atoms. Despite the larger atomic radius of Ca (197 pm) compared to that of Pt (139 pm), the significant difference in their electronegativities (1.00 for Ca and 2.28 for Pt based on the Pauling scale) would lead to a charge transfer when they are alloyed. As Ca tends to lose electrons, and Pt tends to retain or accept electrons, Ca shrinks in size. This charge redistribution makes the effective size of Ca and Pt close to each other. However, as the PtCa alloy is Pt‐rich, lattice mismatch will be developed between the alloy core and Pt‐rich surface leading to strains similar to those observed in Pt‐late transition metals.^[^ ^31^ ^]^

The structure of the acid‐treated PtCa/C catalyst is investigated by XRD (Figure 1e). The PtCa/C matches well with the peaks of closely overlapped moieties of Pt_2_Ca (PDF # 03065174) and Pt_5_Ca (PDF# 03065665) evidenced by the unique appearance of peaks at 20.24, 39.42, 41.26, 48.10, 52.64, 63.90 and 85.00, 2 theta angles. This crystal arrangement corresponds to cubic and hexagonal structures with space groups Fd‐3m (227) and P6/mmm (191), which have never been explored and reported in NP form. Previous works show polycrystalline electrodes of Pt_5_Ca which presents the hexagonal CaCu_5_‐type structure.^[^ ^25^, ^35^ ^]^ We postulate that minor CaO formed during the initial process (Figure S8b, Supporting Information) protects the Pt particles at the high annealing temperature. Otherwise, severe aggregation of the particles would occur. This is similar to previous reports where different groups employed MgO, ZnO, and SiO_2_ as robust protectors against NP aggregation.^[^ ^14^, ^36^, ^37^ ^]^ These oxides facilitate reactant diffusion at high temperatures but limit particle‐to‐particle aggregation. In particular, STEM and the corresponding EDS elemental mapping images after annealing at 850 °C (before acid‐etching) support this postulate as evidenced in Figure S9 (Supporting Information), where the area for Ca and O is the same but larger than that of Pt, with the Pt signal mainly centered at the core of the scanned particle (Figure S9, Supporting Information).

**Figure 2:**
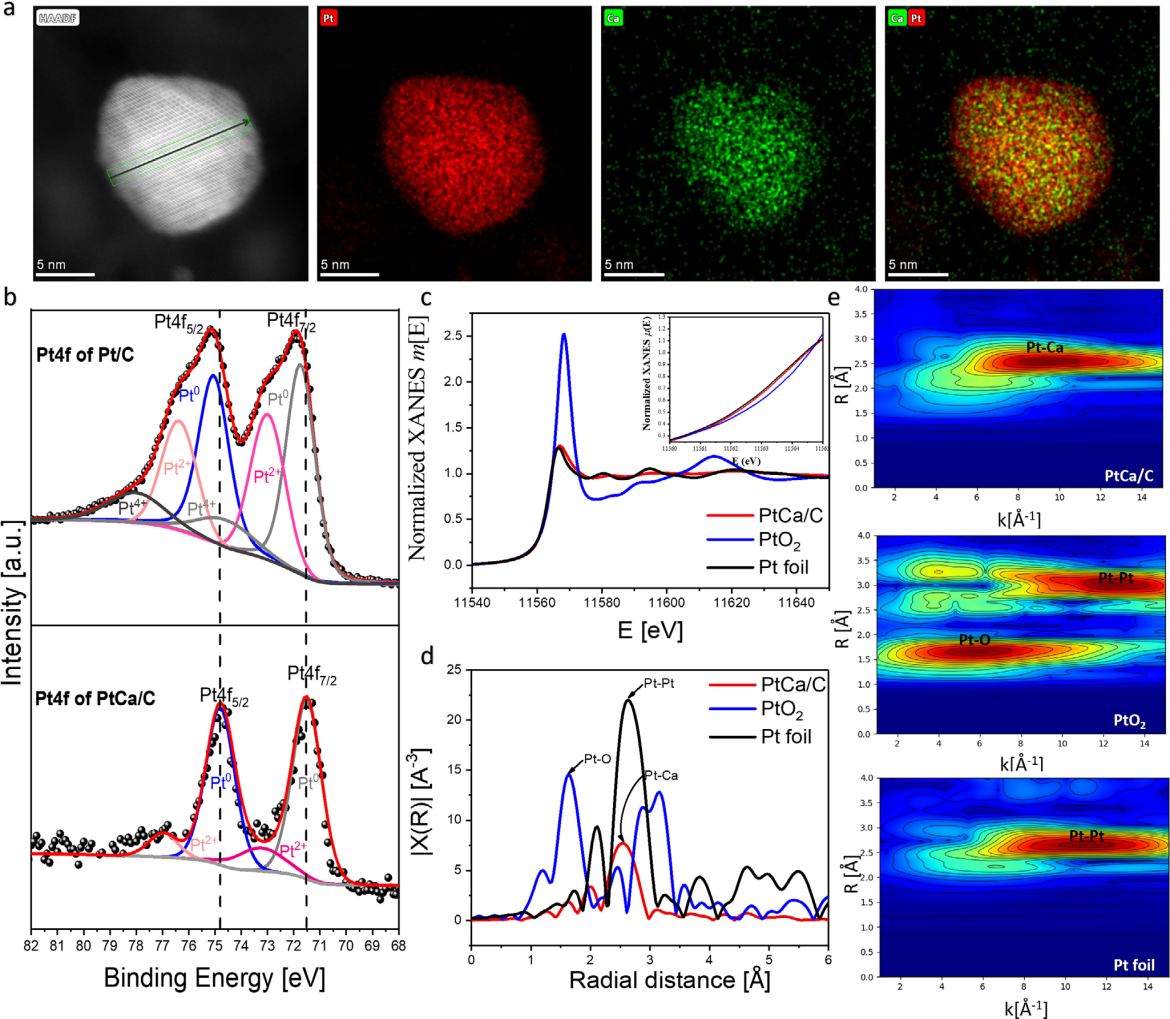
a) HAADF‐STEM image of the particle in “1d” and the corresponding EDS elemental mapping showing Pt (red), Ca (green), and the overlap. b) high‐resolution Pt 4*f* XPS profiles of PtCa/C and commercial Pt/C and c) Pt *L*_3_ XANES and d) Pt *L*_3_ K^2^ – weighted EXAFS spectra of PtCa/C, Pt foil, and PtO_2_ and the corresponding e) wavelet transform (WT) spectra.

The EDS elemental mapping of the HAADF‐STEM images in Figure 1f clearly shows that the Pt area is larger than that of the Ca and that the Ca is mainly concentrated in the core, which is surrounded by a Pt‐rich shell. Typically, in **Figure** **2**a the area corresponding to Pt is larger than that of the Ca, with most of the Ca concentrated at the center of the particle. This Pt outer‐layer created provides kinetic stability by protecting the less noble metal (Ca) against dissolution in the strong electrolyte conditions of PEMFC. Also, the sub‐surface Ca atom underneath the Pt layer acts as a ligand by modifying the surface Pt electronic properties owing to the wide electronegativity difference between Pt (2.28) and Ca (1.00).^[^ ^28^, ^32^ ^]^ This Pt‐rich layer is further corroborated by the HAADF‐STEM and corresponding gray‐scale image in Figure S7 (Supporting Information). TEM and XRD characterization before and after annealing provides some insight into the formation mechanism of the PtCa alloys. Figure S8a (Supporting Information) shows the morphology of the particles heated at 165 °C before annealing at a high temperature. The particles are very small with an average diameter of ≈3 nm. As confirmed by the XRD (Figure S8b, Supporting Information), the peaks are well resolved and follow the pattern for Pt references evidenced by the appearance of (111), (200), (220), and (222) peaks. However, when compared to the Pt reference peak, the (111) peak is slightly shifted to higher angles, and no crystalline peaks associated with CaCl_2_ or Ca can be observed, suggesting that Ca remains amorphous or is doped on the Pt surface. Also, other satellite peaks are present indicating the presence of some reaction intermediates.

STEM‐EDS mapping and elemental composition of the as‐synthesized particles prepared at 165 °C show 73% atomic percent of Ca and 27% Pt (Figure S10, Supporting Information). This ratio conforms with the initial precursor mole ratios of 3:1. This result also suggests that calcium reacts in some way, otherwise the calcium chloride would be leached off during washing. The initial positive shift in the peak position in Figure S8b (Supporting Information) can also be attributed to the trace amounts of CaO formed or the formation of intermediate hydrides i.e. CaH_2_ as proposed in the synthesis of Pt_3_Y,^[^ ^31^ ^]^ Pt_3_V, and Pt_3_Ti.^[^ ^27^ ^]^ This positive shift further suggests that crystalline Pt is formed at the initial reaction temperature of 165 °C. However, the peak position does not reflect the 39.76° 2 theta value usually observed for Pt (111) diffraction planes, emphasizing that doped Ca species may exist on the crystalline Pt surface.

We have also explored other methods of producing PtCa by adopting the method reported earlier in the synthesis of PtxMg alloy.^[^ ^28^ ^]^ In short, we add a pre‐weighed amount of calcium granules obtained from Alfa Aesar to 20 wt% Pt/C prepared as reported in the reference.^[^ ^28^ ^]^ Figure S11a,b (Supporting Information), shows the XRD patterns of the as‐prepared sample at 900 °C (since the melting point of Ca is 842 °C). From these XRD patterns (Figure S11a, Supporting Information), CaO peaks are identified, which can be ascribed to the surface oxidation of calcium granules upon exposure to air. The XRD profile before acid etching also shows a positive shift to higher angles relative to Pt reference peaks. We attribute this observation to the interaction between the CaO and Pt particles. Upon washing with 1.0 M HCl, no CaO peaks are detected, and the (111) peak position of the as‐synthesized sample shifts to a 2‐theta value of 39.8°. This observation depicts that no substantial alloy formation occurs using this method. This control experiment suggests that the wet chemistry approach employed in our synthesis is more efficient as compared to the top‐down approach previously utilized.

The compositional and line scanning profiles in Figure 1f and corresponding EDS elemental mapping (Figure 2a; Figure S12, Supporting Information) collectively reveal that the Pt and Ca are uniformly distributed throughout the particles, further confirming the alloy formation. The elemental composition of the acid‐treated sample as determined from the EDS scan reveals Pt to Ca atomic percentage of 65.77: 34.23 (Figure S13; Table S2, Supporting Information). Despite the coexistence of both Pt_2_Ca and Pt_5_Ca phases, the EDS composition of Pt to Ca is closer to the stoichiometric value of Pt_2_Ca. The chemical compositions and electronic structure of the PtCa/C are further characterized by XPS. The XPS survey spectrum (Figure S14a, Supporting Information) shows the presence of both Pt and Ca with a surface composition of 57.18: 42.82 atomic % for Pt and Ca respectively. This value for Ca is slightly higher compared to the value observed in the EDS spectrum. This difference in composition with respect to TEM‐EDS and XPS can be attributed to the difference in detection limits associated with the two characterization techniques. This observation is also in line with STEM‐EDS maps in Figures 2a and S12 (Supporting Information), where the Pt is only present in the NP area, whereas the Ca signal is detected across the entire area of the catalyst, with the strongest signal located at the core of the NP and the weaker signal on the carbon substrate. This observation infers that there might be the existence of Ca atomic clusters too small to be detected by the probe‐corrected STEM. To confirm the actual Pt:Ca atomic ratio, inductively coupled plasma optical emission spectroscopy is employed. The results show a Pt to Ca ratio of 60.84 to 39.16, respectively.

High‐resolution Pt 4*f* XPS spectra for both PtCa/C and commercial Pt/C are illustrated in Figure 2b. The spectra for both samples consist of two main doublets; Pt 4*f*_5/2_ and Pt 4*f*_7/2_. In all cases, there is a negative shift in binding energies of the Pt 4*f* doublets of PtCa/C relative to that of commercial Pt/C. The Pt (0) peak positions are located at binding energies of 74.8 and 71.5 eV for the Pt4*f*_5/2_ and Pt4*f*_7/2_ peaks, respectively for PtCa/C while those of commercial Pt/C are located at binding energies of 75.1 and 71.76 eV. The negative shift of ≈0.27 eV in both doublets is an indication of strong ligand and strain effects between the Pt and Ca as a result of the electronegativity difference between the two metals.

XAS is further utilized to affirm the impact of Ca on the alloy structure. X‐ray absorption near edge structure (XANES) (Figure 2c (insert)) shows that the absorption edge of the PtCa alloy slightly shifts to positive energy relative to that of the Pt foil but is substantially distant from that of the PtO_2_, suggesting an electron cloud shift from the Ca atoms to the Pt atoms. The corresponding Fourier transformed extended X‐ray absorption fine structure (EXAFS) of the high energy regions in R‐space (Figure 2d) shows that the Pt─Pt bond distance is longer than the Pt─Ca bond distance possibly due to the large electronegativity differences between the two metal atoms. Such a difference in electronegativity could induce a significant ionic character, which would lead to a significant decrease in the ionic radius of Ca and the induction of compressive strains.^[^ ^32^ ^]^ Furthermore, the substantial difference in atomic radius could also induce strains along different crystallographic directions. These two effects probably account for the shorter Pt─Ca bond length observed in the XAS data. The fitting results are shown in Figure S15 and further summarized in Table S3 (Supporting Information). The PtCa/C is determined to have a 20.4 wt% metallic component as recorded by a thermogravimetric analyzer (TGA) in Figure S16 (Supporting Information).

### 2.2 Electrochemical Half‐Cell and MEA Full‐Cell Test

**Figure 3:**
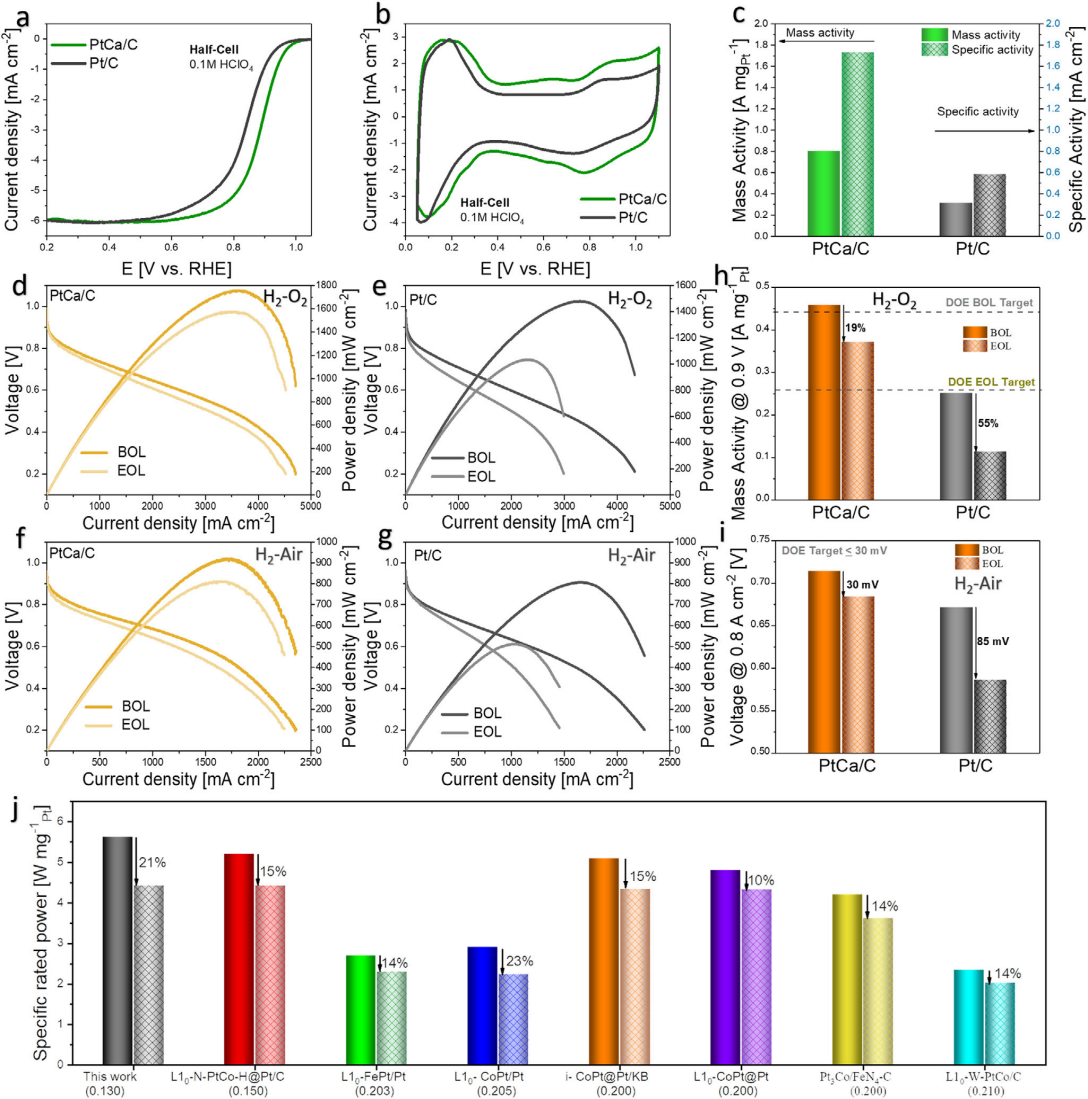
Half‐cell and Full‐cell MEA test. a) LSVs conducted in O_2_‐saturated 0.1 M HClO_4_, b) CVs conducted in N_2_‐saturated 0.1 M HClO_4_. c) Histograms of mass and specific activities recorded before and after 30k ADT. d and e) H_2_‐O_2_ fuel cell polarization curves and power density curves of PtCa/C and Pt/C before and after 30k ADT. f and g) H_2_‐air fuel cell polarization curves and power density curves of PtCa/C and Pt/C before and after 30k ADT. h) Changes in mass activities (MAs) for the two catalysts before and after 30k ADT under H_2_‐O_2_ conditions. i) Voltage drops @ 0.8 A cm^−2^ for PtCa/C and Pt/C before and after 30k ADT. j) Specific rated power of PtCa/C at 0.67 V under H_2_‐air atmosphere and combined anode‐cathode Pt loading compared to recently published high‐performing PEMFC electrocatalysts.^[^ ^17^, ^38^, ^39^, ^40^, ^41^, ^42^, ^43^ ^]^

The electrocatalytic property of the PtCa/C is evaluated in half‐cell using a three‐electrode system in 0.1 M HClO_4_ with an Ag/AgCl electrode as the reference electrode, a Pt wire as the counter electrode, and a glassy carbon as the working or indicator electrode (Details in Supporting Information). The electrochemical performance is subsequently compared to commercial Pt/C (19.4 wt% Tanaka Kikinzoku Kogyo, Japan). In **Figure** **3**a, PtCa/C exhibits a half‐wave potential of 0.87 V (iR‐correction free), 35 mV higher in comparison to commercial Pt/C. This demonstrates that the PtCa/C has enhanced catalytic activity compared to the Pt/C. The cyclic voltammograms (CVs) in Figures 3b and S17a (Supporting Information) show that the oxide‐hydroxide adsorption peak of PtCa/C is shifted to a more positive potential relative to commercial Pt/C. This observation can be attributed to the strong alloy formation between Pt and Ca which tends to weaken the Pt─O binding strength. A similar observation can be made for the oxide reduction peaks (Figure S17b, Supporting Information) as indicated by the enlarged portion of the CV in Figure 3b. The electrochemically active surface areas (ECSAs) of both Pt/C and PtCa/C are determined through the hydrogen underpotential deposition (HUPD) method (Experimental section). The area corresponding to the HUPD in the CV is slightly lower than that recorded for commercial Pt/C with ECSAs of 47.03 and 53.99 m^2^ g^−1^ for PtCa/C and Pt/C respectively (Figure S18b, Supporting Information). The difference in ECSAs likely emanates from the difference in particle size, as demonstrated by the TEM images in Figure S24 (Supporting Information).

To further quantify the intrinsic ORR activity, we obtain the kinetic current of each catalyst using the Koutecky‐Levich equation (Experimental section). At the potential region of 0.9 V vs reversible hydrogen electrode (RHE), the PtCa/C delivers a half‐cell mass activity (MA) of 0.80 A mg^−1^_Pt_ (Figure 3c). This intrinsic activity is three (3) times higher than commercial Pt/C (0.31 A mg^−1^_Pt_). This value represents one of the best reported for Pt‐alkaline earth metals, Pt‐lanthanides, and Pt‐early transition metals (Table S4, Supporting Information). As reported by Greeley et al.,^[^ ^29^ ^]^ modern PEMFCs have been designed for the efficient delivery of reactive gases, and thus, we are primarily concerned with kinetic transport rather than mass transport. To compare the kinetics, we plot the Tafel slopes (0.8 – 1.00 V vs RHE) for the two catalysts. In Figure S17c (Supporting Information), PtCa/C exhibits a lower Tafel slope of 60.8 mV compared to that of commercial Pt/C with 76.8 mV. The electrochemical durability test in the acidic electrolyte is further assessed by conducting accelerated durability tests. ECSAs are determined after 10k, 20k, and 30k cycles, respectively for the PtCa/C (Figure S18, Supporting Information). From this figure, there is no significant loss in ECSA after 10, 20, and 30k ADT. Although the PtCa/C shows a 19‐mV loss in activity (half‐wave potential) after 30k, this drop is much better when compared to Pt/C measured under the same conditions (Figure S19, Supporting Information). The RDE performance of the PtCa before high temperature annealing (PtCa/C‐165) is indicated in Figure S20 (Supporting Information). The results indicate the high temperature annealing is essential for the PtCa alloy formation which translates to enhanced fuel cell performance.

We also evaluate the performance of the PtCa/C in a practical fuel cell by employing it as the cathode in the membrane electrode assembly (MEA). Figure S21a (Supporting Information) presents the H_2_‐O_2_ polarization curves of PtCa/C and commercial Pt/C. PtCa/C shows a good performance in the kinetic range (438 mA cm^−2^ @ 0.8 V), higher than that of commercial Pt/C (292 mA cm^−2^ @ 0.8 V). The initial peak power density (Figure S21b, Supporting Information) of PtCa/C (1755 mW cm^−2^) is significantly higher than commercial Pt/C (1480 mW cm^−2^). Impressively, the PtCa/C displays a higher stability compared to the commercial Pt/C. The polarization and power density curves of PtCa/C in Figure 3d display only a small change in performance after 30 000 cycles, evidenced by the 10% drop in peak power density. In contrast, the Pt/C displays a loss of 30% (Figure 3e). The PtCa/C reaches a mass activity of 0.45 A mg^−1^_Pt_ [beginning of life (BOL)] and 0.37 A mg^−1^_Pt_ [end‐of‐life (EOL)] at 0.9 V, corresponding to a 19% loss after 30 000 cycles of ADT, surpassing the DOE 2025 target in EOL mass activity (0.26 A mg^−1^_Pt_, <40% of initial mass activity). In disparity, the Pt/C shows a 55% loss in initial mass activity (from 0.25 A mg^−1^_Pt_ (BOL) to 0.11 A mg^−1^_Pt_ (EOL)), a performance drastically below the DOE 2025 target (Figure 3h; Table S4, Supporting Information).^[^ ^17^ ^]^

The fuel cell performance is assessed under H_2_‐air conditions to simulate practical operating scenarios. The polarization curves depicted in Figure S22a (Supporting Information) and the corresponding histogram plots (Figure S22b, Supporting Information) reveal that PtCa/C demonstrates a higher peak power density of 900 mW cm^−2^. Notably, a comparable 11% reduction, similar to that observed under H_2_‐O_2_ conditions, is recorded after 30k ADT (Figure 3f). In contrast, the peak power density of Pt/C experiences a substantial decrease by 37%, dropping from its initial peak power density of 810 to 510 mW cm^−2^ after ADT (Figure 3g). Additionally, PtCa/C meets the criterion of a voltage drop below 30 mV at 0.8 A cm^−2^ after ADT, as mandated by the US DOE, with a 30‐mV drop compared to the 85‐mV drop observed for Pt/C (Figure 3i). The specific rated power is determined to be 9 and 5.3 W mg^−1^_Pt_ at 0.67 V for cathode and combined anode & cathode loadings respectively, under H_2_‐air conditions (Figure 3J; Figure S23, Supporting Information). These values signify exceptional catalytic performance, ranking among the best compared to recently published highly active cathode catalysts for fuel cells.

In addition, the Pt/C shows increased aggregation from 2 to 5–7 nm, while the PtCa/C exhibits only a slight increase from 6 to 8 nm (Figure S24, Supporting Information). The STEM‐EDS survey spectrum shows a loss of 35% in Ca composition (from 34% to 22%) (Figures S25 and S26, Supporting Information), emphasizing the enhanced stability achieved by alloying Ca with Pt. Interestingly, the HAADF‐STEM image presented in Figure S27 (Supporting Information) evinces that the PtCa largely retains its ordered structure after the 30k ADT. The excellent stability exhibited by the PtCa/C places it among the most competitive Platinum‐Alkaline, Platinum‐Rare Earth, and Platinum‐Transition metal ORR catalysts reported to date (Table S4, Supporting Information). This gives us confidence that the PtCa/C can provide great potential in transportation applications. Besides the activity and stability, the conductivity of the electron and proton in the catalyst layer is of great importance in fuel Cells. Here, electrochemical impedance spectroscopy is employed to study the characteristics of the various MEAs in terms of conductivity and charge transfer at the catalyst‐electrolyte interface. An equivalent circuit is considered to study the impedance behavior in the catalyst's layers (Figure S28c, Supporting Information). The Nyquist plots for commercial Pt/C and PtCa/C are shown in Figure S28a (Supporting Information). From this data, the impedance spectra of the two samples display similar characteristics, i.e. a depressed semi‐circle in the high‐frequency region. The depressed semi‐circle in the high‐frequency region is ascribed to the charge (electron and proton) exchange at the catalyst/electrolyte interface. As observed in Figure S28a,b (Supporting Information), both catalysts display similar charge transfer properties at the beginning of life. After 30 000 cycles, however, the charge resistance of commercial Pt/C increases by 79% while that of the PtCa/C only increases by 17%, emphasizing the durable nature of the PtCa/C.

### 2.3 Theoretical Studies

To gain insight into the activity and stability trends of Pt and Pt─Ca model systems under ORR conditions, we apply electronic structure theory calculations in the density functional theory (DFT) framework. All computational details are listed in the Supporting Information, Notes S1 and S2 (Supporting Information) (Computational details, Pt─Ca system, Modeling of electrochemical processes). There, we also describe our approach to the modeling of the Pt─Ca system, which is based on the substitution of every second Pt atom by Ca, except for the top layer, to resemble the core‐shell concept (Figure S29, Supporting Information).

For the investigated Pt(111) and PtCa(111) surfaces, we solve the ORR mechanism and the limiting step by combining free‐energy diagrams with a descriptor‐based analysis.^[^ ^44^ ^]^ Our mechanistic analysis considers the effect of surface coverage under ORR conditions, which has been shown to be crucial for modeling catalytic processes under applied bias.^[^ ^45^ ^]^ To this end, we construct a surface Pourbaix diagram^[^ ^46^, ^47^ ^]^ using DFT calculations for the Pt(111) surface,^[^ ^48^ ^]^ which is discussed in Note S3 (Supporting Information) (Construction of surface Pourbaix diagrams) of the SI. The Pourbaix diagram reveals that a fully hydroxylated surface (Figure S30, Supporting Information) – Pt(111)‐9*OH– is thermodynamically stable under ORR conditions. Therefore, the investigation of activity and stability trends is carried out using the Pt(111)‐9*OH and PtCa(111)‐9*OH surface configurations (Figure S29, Supporting Information).

**Figure 4:**
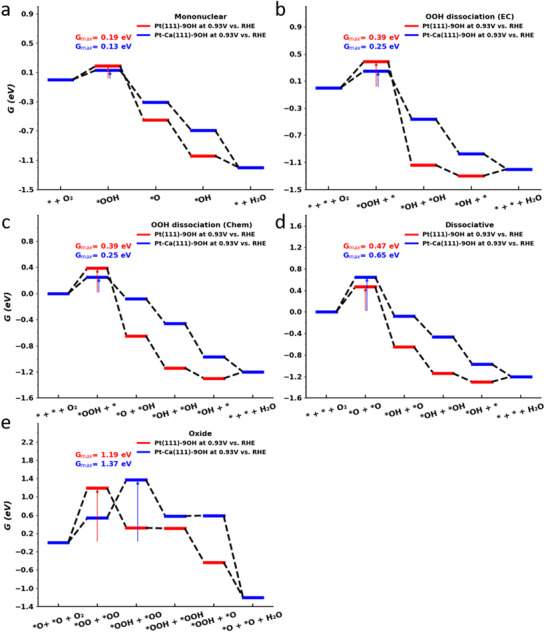
Free‐energy landscapes for five ORR mechanisms at *U* = 0.93 V vs. RHE on Pt(111)‐9*OH (red) and PtCa(111)‐9*OH (blue). a) Mononuclear mechanism, b) OOH dissociation mechanism by an electrochemical (EC) step, c) OOH dissociation mechanism by a chemical (Chem) step, d) Dissociative mechanism, and e) Oxide mechanism.

ORR is a complex four‐proton coupled electron transfer process, and the conversion of one oxygen molecule into two water molecules can occur through different mechanistic pathways.^[^ ^49^, ^50^, ^51^ ^]^ In Note S4 (Supporting Information) (Modeling of ORR mechanisms) of the SI, we summarize five different mechanistic pathways that are considered in this work, ranging from mononuclear, dissociative, oxide, and two different OOH dissociation descriptions.^[^ ^52^, ^53^, ^54^ ^]^ **Figure** **4** illustrates the free‐energy landscapes for these five ORR mechanisms over the Pt(111)‐9*OH and PtCa(111)‐9*OH surfaces at U = 0.93 V vs RHE, corresponding to an ORR overpotential of 300 mV. To estimate the catalytic activity, we analyze these free‐energy diagrams by using the descriptor G_max_(U),^[^ ^55^ ^]^ which is a representation of the energetic span model for electrocatalytic processes.^[^ ^56^, ^57^ ^]^

Among the pathways considered, the mononuclear mechanism turns out to be the preferred mechanistic description for both Pt(111)‐9*OH and PtCa(111)‐9*OH due to the lowest G_max_(U = 0.93 V) values. This suggests that Ca doping does not alter the reaction pathway but rather affects the relative stability of the intermediate states. A comparison of the electrocatalytic activity indicates a slight enhancement in the ORR kinetics for the Ca‐doped Pt surface due to a lower G_max_(U = 0.93 V) value (0.13 eV) compared to Pt (0.19 eV). Note that a smaller free‐energy span is associated with faster reaction kinetics under the same electrochemical conditions.^[^ ^55^ ^]^

Despite the improved catalytic performance, the limiting reaction step in the framework of the descriptor G_max_(U) remains unchanged when moving from Pt to PtCa. In both cases, the descriptor G_max_(U) is governed by the first elementary step – * + O_2_ → *OOH + (H^+^ + e^−^) – which is consistent with previous work by Huang and coworkers on this topic.^[^ ^58^ ^]^ While the formal reaction kinetics do not change when Ca is introduced in the core of the Pt catalyst, the substitution of Pt by Ca in the subsurface layers modifies the electronic structure of the Pt surface atoms, which, in turn, influences the adsorption free energies of key intermediates such as *OOH, *O, and *OH in the ORR. We attribute the reduced G_max_(U = 0.93 V) value of PtCa compared to Pt to an improved stabilization of the *OOH intermediate, which, considering the Brønsted‐Evans‐Polanyi relationship,^[^ ^59^ ^]^ leads to faster adsorption of the reactant O_2_ compared to Pt.

**Figure 5:**
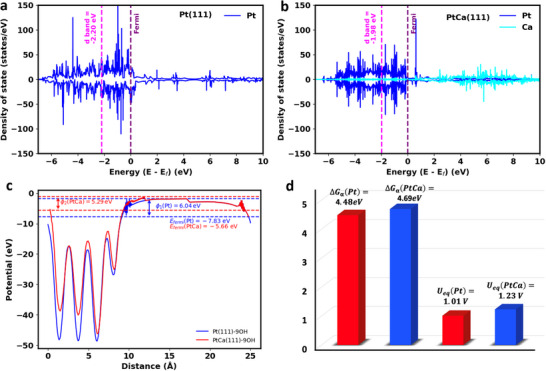
a) Projected density of states (PDOS) for the bare Pt(111) surface, with a d‐band center energy of ‐2.20 eV (E_fermi_ = ‐7.63eV), and b) PDOS for the bare PtCa(111) surface, with a d‐band center energy of ‐1.98 eV (E_fermi_ = ‐5.17eV). c) Variation of the local potential and the calculated work functions Φ1(Pt) = 6.04eV and Φ2(PtCa) = 5.29 eV for Pt(111)‐9*OH and PtCa(111)‐9*OH, respectively. d) Catalysis stability analysis: Variation of ΔGα (eV) and Ueq (V) for both Pt(111) and PtCa(111).

To gain further insight into the electronic structure modification of Pt/Ca substitution, we calculated the projected density of states (PDOS) and work functions for the bare Pt(111) and PtCa(111) surfaces to determine their d‐band center^[^ ^60^ ^]^ in **Figure** **5**a–c. For the Pt(111) surface, the d‐band center is located at −2.20 eV, which is in line with previous work by Adzic and coworkers.^[^ ^61^ ^]^ In contrast, the d‐band center for the PtCa(111) surface is shifted to a slightly higher energy of −1.98 eV. This rightward shift in the center of the d‐band compared to Pt suggests that the electronic environment of the Pt atoms is influenced by the presence of Ca atoms below the surface. Interestingly, the single‐crystal experiments by Adzic and coworkers indicate that a shift of the Pt d‐band center to the right by ≈0.3 eV should result in an ORR catalyst with optimum electrocatalytic activity.^[^ ^61^ ^]^ We find that this criterion is obviously met by the introduction of Ca into the core of the Pt catalyst and therefore attribute the improved electrocatalytic activity of PtCa compared to Pt to the shift in the center of the d‐band. Please note that the rightward shift in the center of the d‐band for PtCa(111) is also observed if adsorbate coverage is considered in the DFT calculations, which is discussed in Note S5 (Supporting Information) (Density of states) of the (Figure S31, Supporting Information).

Finally, we evaluate the stability of Pt and PtCa catalysts under applied bias by employing a Born‐Haber cycling approach.^[^ ^62^ ^]^ While the detailed methodology of our procedure is described in Note S6 (Supporting Information) (Catalysis stability analysis) of the SI, our approach aims to derive an equilibrium potential for the leaching of a Pt atom from the catalyst surface into the electrolyte under anodic conditions, and a higher equilibrium potential indicates improved stability under anodic polarization. While for Pt(111)‐9*OH the equilibrium potential for Pt dissolution is determined to be 1.01 V (Figure 5d), we obtain 1.23 V for PtCa(111)‐9*OH. Therefore, we conclude that PtCa exhibits greater resistance to Pt leaching, suggesting that the incorporation of Ca into the core improves the stability of the catalyst under ORR conditions, which is in line with the experimental data (Figure 3). The reason for the improved stability of PtCa compared to Pt is related to the fact that vacancy formation, ΔGα, is energetically more demanding in PtCa than in Pt (Figure 5d).

## 3 Conclusion

In this work, we report for the first time the synthesis of PtCa alloy NP catalyst via a nonaqueous solution phase under air‐free conditions. Contrary to previous reports on polycrystalline Pt_5_Ca electrodes, the current PtCa catalyst is present in the form of nanoparticles. The PtCa exhibited superior activity and much‐enhanced stability when employed as a cathode catalyst in both half‐cell and full cell. Specifically, in full Cell PEMFC test, it exhibited only a 10% drop in peak power density and a 19% drop in mass activity after 30k ADT according to the US DOE protocol, representing just one of the two platinum‐alkaline earth catalysts to report PEMFC performance. Furthermore, the specific rated power of 9 W mg^−1^_Pt_ represents one of the best performances reported to date. Another key observation is that PtCa breaks the typical activity‐stability trade‐off often encountered in catalyst design. While catalysts with higher activity tend to be less stable, our density functional theory calculations reveal that PtCa exhibits both improved ORR activity and higher stability compared to Pt. We attribute the higher activity to a shift of the d‐band center while the reaction kinetics remain unchanged, and the improved stability is explained by the fact that the formation of vacancies due to Pt dissolution is energetically unfavorable for the Ca‐substituted catalyst. We believe that this catalyst holds significant promise as an active and durable catalyst in PEMFC for heavy‐duty applications.

## Conflict of Interest

The authors declare no conflict of interest.

## Supporting information



Supporting Information

## Data Availability

The data that support the findings of this work can be found in the Supporting Information. Further inquiries can be made to the corresponding author upon reasonable request.
