# Supplementary material for: Intermetallic Platinum‐Calcium Alloy Breaks the Activity‐Stability Trade‐Off in Fuel Cell for Enhanced Performance
Source: Small. 2025 Jun 5;21(31):2503692. doi: 10.1002/smll.202503692 (PMC12332820; doi:10.1002/smll.202503692)
Supplement: Supplementary file 1 — Supporting Information [file SMLL-21-2503692-s001.docx]

Supporting Information

Intermetallic Platinum-Calcium Alloy Breaks the Activity-Stability Trade-off in Fuel Cell for Enhanced Performance

*Caleb Gyan-Barimah,+ Kapil Dhaka,+ Ha-Young Lee, Yi Wei, Muhammad Irfansyah Maulana, Jeong-Hoon Yu, Bo Yu, Kai S. Exner,* and Jong-Sung Yu**

Corresponding Authors: [kai.exner@uni-due.de](mailto:kai.exner@uni-due.de); jsyu@dgist.ac.kr

Table of Contents

[Experimental Procedures 5](#_Toc197601213)

[Chemicals/Reagents 5](#_Toc197601214)

[Synthesis of PtCa nanoparticles 5](#_Toc197601215)

[Alternative method 5](#_Toc197601216)

[Characterization. 5](#_Toc197601217)

[Electrochemical measurements. 5](#_Toc197601218)

[MEA Preparation and fuel Cell testing 6](#_Toc197601219)

[Computational details 6](#_Toc197601220)

[Figure S1. a and b) TEM images of PtCa/VC annealed at 850 0C on Vulcan carbon (VC) as support. Scale bars for a and b are 100 and 10 nm respectively. c) HAADF-STEM image of selected PtCa NPs and d, e, and f) corresponding EDS elemental mappings (Scale bars are in 100 nm). 7](#_Toc197601221)

[Figure S2. XRD patterns of the PtCa catalyst obtained after 850 oC annealing treatment. The blue and red XRD patterns represent the sample before (BE) and after acid treatment respectively 8](#_Toc197601222)

[Figure S3. a) TEM image, b) XRD patterns, and high resolution XPS spectra of c) Y 3d and d) Pt 4f scan of Pt3Y/C. 9](#_Toc197601223)

[Figure S4. XRD patterns of PtSr/C synthesized at 900 oC. 10](#_Toc197601224)

[Figure S5. XRD patterns of PtBa/C synthesized at 900 oC. Peaks with * corresponding to pure Pt diffraction peaks. 11](#_Toc197601225)

[Figure S6. a) TEM image of PtCa/C and (b) histogram of the corresponding particle size distribution. 12](#_Toc197601226)

[Figure S7. (a) HAADF-STEM image of the PtCa alloy annealed and acid-etched after the 850 oC annealing treatment and (b) the corresponding gray-scale image showing the Intermetallic core, Pt-rich shell arrangement. 13](#_Toc197601227)

[Figure S8. a) TEM image of PtCa/C as prepared by heating at 165 oC for 24 h before annealing at high temperature (850 oC). b) XRD patterns of as-prepared catalyst for 12 h (black) and 24 h (red) of reaction. 14](#_Toc197601228)

[Figure S9. HAADF-STEM image and the corresponding EDS elemental mapping images of PtCa/C prepared at 850 oC before acid etching. The area corresponding to Ca (green) and O (turquoise blue) is larger than that of Pt (red), where Pt is mainly centered in the core of the particle, evincing the presence of CaO protection. 15](#_Toc197601229)

[Figure S10. a) STEM image and the corresponding EDS mappings for b) Pt c) Ca and d) EDS survey spectrum and elemental composition of as-prepared PtCa catalyst treated at 165 oC for 24 h. 16](#_Toc197601230)

[Figure S11. a) XRD patterns for PtCa/C-Powder synthesized at 850 oC using PtxMg approach before(red) and after acid etching (blue). b) Pt (111) XRD peak shift for PtCa/C-Powder. 17](#_Toc197601231)

[Figure S12. Large area STEM image and corresponding EDS elemental mapping of PtCa//C NPs showing C (blue), Pt (red) and Ca (green). 18](#_Toc197601232)

[Figure S13. EDS Survey spectrum of PtCa/C catalyst annealed at 900 0C with an atomic composition of 65.77% for Pt and 34.23% for Ca. 19](#_Toc197601233)

[Figure S14. a) XPS survey spectrum and high-resolution XPS spectra b) Ca 2p, c) Ca 2s of the PtCa/C sample. 20](#_Toc197601234)

[Figure S15. Extended X-ray absorption fine structure (EXAFS) analysis. Experimentally obtained data (red line) and the fitting results (white circles) of the Fourier transformed EXAFS spectra of PtCa/C, Pt foil, and PtO2 in a, c, and e) R-space and b, d, and f) K-space, respectively. 21](#_Toc197601235)

[Figure S16. TGA data recorded for the PtCa/C. 22](#_Toc197601236)

[Figure S17. a and b) The Enlarged portion of the CV in Figure 3b showing the difference in potential between Pt oxidation and reduction peaks and c) Tafel plots for PtCa/C and Pt/C. 23](#_Toc197601237)

[Figure S18. a) CV’s recorded in O2 saturated 0.1 M HClO4 at a scan rate of 50 mV s-1 for PtCa/C before and after 10k, 20k, and 30k of ADT test b) histograms of the ECSA at BOL for Pt/C and PtCa/C and c) PtCa at BOL, after 10k, 20k and 30k EOL, respectively. 24](#_Toc197601238)

[Figure S19. Linear sweep voltammetry (LSV) curves of a) PtCa/C and b) Pt/C before and after 30,000 ADT. 25](#_Toc197601239)

[Figure S20. a) Linear sweep voltammetry (LSV) curves and b) cyclic voltammograms (CVs)s of PtCa/C-165, PtCa/C and Pt/C. The PtCa/C-165 represents the sample prepared at 165 oC before the high temperature annealing. 26](#_Toc197601240)

[Figure S21. a) H2-O2 fuel cell polarization curves of commercial Pt/C and PtCa/C recorded before ADT at 0.5 bar back pressure and 100% RH. b) The histogram of the peak power densities before and after 30 k ADT recorded under the same conditions. 27](#_Toc197601241)

[Figure S22. a) H2-air fuel cell polarization curves of commercial Pt/C and PtCa/C recorded before ADT at 0.5 bar back pressure and 100% RH. b) The histogram of the peak power densities before and after 30 k ADT recorded under the same conditions. 28](#_Toc197601242)

[Figure S23. Specific rated power of PtCa/C compared to recently published high-performing PEMFC electrocatalysts.[14–22] 29](#_Toc197601243)

[Figure S24. TEM images before and after 30k ADT respectively for a and b) commercial Pt/C c and d) PtCa/C. 30](#_Toc197601244)

[Figure S25. HAADF-STEM image and the corresponding EDS of a single PtCa after 30 k ADT. 31](#_Toc197601245)

[Figure S26. EDS spectrum for PtCa/C after 30k ADT test. 32](#_Toc197601246)

[Figure S27. HAADF-STEM images of PtCa particles after 30 k ADT. 33](#_Toc197601247)

[Figure S28. a) PEMFC Electrochemical impedance spectra (Nyquist plots) and b) charge transfer resistance (Rct) changes for PtCa/C and Pt/C before and after 30k potential cycling. c) Equivalent circuit employed for the measurement of the impedance. 34](#_Toc197601248)

[Figure S29. Depiction of a stable fully hydroxylated surface (9 *OH, cf. Pourbaix diagram in Figure S26) of Pt(111) and PtCa(111). In the PtCa(111) configuration, Ca is doped in the subsurface layers of the slab, which resembles the core-shell concept, with Pt serving as the active site for ORR. 35](#_Toc197601249)

[Note 1: Pt-Ca system 35](#_Toc197601250)

[Note 2: Modeling of electrochemical processes 36](#_Toc197601251)

[Figure S30. Pourbaix diagram for Pt(111) obtained from electronic structure theory calculations in the DFT framework.[27] A fully hydroxylated surface – 9 *OH – is observed under typical ORR conditions (U = 0.93 V vs. RHE). 37](#_Toc197601252)

[Note 3: Construction of surface Pourbaix diagrams 37](#_Toc197601253)

[Note 4: Modeling of ORR mechanisms 38](#_Toc197601254)

[Figure S31. Projected density of states (PDOS) for a) Pt(111)-9OH surface, with a d-band center energy of –2.85 eV, and b) PtCa(111)-9OH surface, with a d-band center energy of –2.80 eV. 39](#_Toc197601255)

[Notes 5: Density of states: 39](#_Toc197601256)

[Note 6: Modeling of catalyst stability 40](#_Toc197601257)

[Table S1. Free energies for the Born-Haber Cycle of equations (eq30-35) 41](#_Toc197601258)

[Table S2. Elemental Composition determined from EDS and XPS. 42](#_Toc197601259)

[Table S3. Structural information and fitting parameters obtained from Pt L3-edge EXAFS spectra of Pt Foil, PtCa/C, and PtO2. (S02 = 0.8426). 43](#_Toc197601260)

[Table S4. ORR Half-Cell and Full-Cell Mass activity and durability of reported Pt-alkaline earth, Pt-lanthanide, Pt-early transition alloys. 44](#_Toc197601261)

**Experimental Procedures**

**Chemicals/Reagents**

Hydrochloric acid (HCl, 35-37%), Platinum (IV) Chloride (PtCl_4_, > 99.9%), Calcium chloride (CaCl_2_, > 97.0%), Magnesium chloride (MgCl_2_ > 98%), Yttrium (III) chloride (YCl_3_ > 99.9%), Strontium choride (SrCl_2_ > 99.9%), Barium Chloride (BaCl_2_ > 99.9%), Sodium Borohydride (NaBH_4_ > 98.0%), Potassium triethylborohydride (KEt_3_BH), Lithium triethylborohydride (LiEt_3_BH), Ascorbic Acid (C_6_H_8_O_6_), Glucose (C_6_H_12_O_6_, > 99.5%), Phloroglucinol (C_6_H_6_O_3_, > 99.0%), N-N Dimethylformamide (DMF), and 5wt% Nafion ionomer solution were all purchased from Sigma-Aldrich Korea. Pt-solution (100 mg ml^-1^) was purchased from Korea, Calcium granules from Alfa Aesar Korea, Commercial Pt/C (20wt. % Pt) from Tanaka Kikinzoku Kogyo, Japan, Vulcan Carbon (Vulcan XC72) from Cobot, and commercial carbon (Ketjen Black EC600JD, BET surface area 1270 m^2^g^-1^) from Ketjen Black international. All chemicals were used as received without further treatment and all water used in the experiment was made by passing through an ultrapure purification system.

**Synthesis of PtCa nanoparticles**

PtCa nanoparticles were prepared by a simple solvo-thermal method using DMF as solvent. In a typical synthesis, 0.076 mmol of Platinum (IV) chloride (PtCl_4_), 0.340 mmol of calcium chloride (CaCl_2_), and 4.758 mmol of NaBH_4_ were dissolved into 15 mL pre-degassed DMF in a vial under inert conditions in an argon-filled glove box. DMF was chosen for its low boiling point (153 ^o^C), enabling faster collisions at lower temperatures, while protic solvents were undesirable due to Ca’s low reduction potential. To minimize oxygen excess, DMF was degassed and stored in an argon-filled glove box before use. After the vial was capped and sealed with Teflon tape, the mixture was ultra-sonicated for 2 hours using a portable ultrasonic cleaner (JAC Ultranosonic- JAC-3010) with an ultrasonic frequency of 40 kHz and ultrasonic power of 300 W. The resulting mixture was transferred into a Teflon-lined autoclave (25 mL) and heated from room temperature to 165 ^0^C. The temperature was maintained at 165 ^0^C for time 12 and 24hrs respectively. The reaction mixture was allowed to naturally cool to room temperature and the resulting colloidal product was collected by centrifugation. The obtained catalyst was collected and deposited on 80 mg of commercial carbon (Ketjen Black EC 600 J) and mixed with a small amount of dry powder of Potassium Triethylborohydride. Subsequently, heat treatment was performed in 5% H_2_/Ar at 850 ^0^C for 4hrs, followed by acid etching in 1M HCl for 5 hours at 70 ^0^C. The final product was collected for further characterization.

**Alternative method**

This is a modification to the previous synthesis of Pt_x_Mg.^[1]^ In this method, Aqueous Pt-solution and 80 mg of pretreated commercial carbon are mixed to give Pt/C with 20wt% for Pt. The mixture was sonicated for 30 min and then heated under magnetic stirring to evaporate water until a thick slurry was formed. The slurry was dried in a vacuum oven and subsequently transferred into a glove box. In the glove box, 13 mg of calcium granule was weighed and mixed with the As-prepared 20 wt% Pt/C and grounded in a mortar to achieve an even distribution of calcium particles. The mixture was transferred from an air-tight vial to a tube furnace saturated with Argon gas. The powdered mixture was subsequently annealed in a tube furnace under a mixture gas (5% H_2_/Ar) flow of 200 ml/min at 850 ^0^C. The obtained product was etched with 1M HCl for 5 hours and further characterized.

**Characterization.**

Powder X-ray diffraction (XRD) was recorded using a Rigaku Smartlab diffractometer with Cu-Kα (0.15406nm) operated at 40 kV and 30 mA at a scan rate of 10 ^o^min^-1^. X-ray photoelectron spectroscopy (XPS) measurements were obtained using an AXIS NOVA (Kratos) X-ray photoelectron spectrometer using a monochromated Al Kα X-ray source (hv = 1486.6 eV) operated at 150 W under a base pressure of 2.6 x 10 ^-9^ Torr. The XPS spectra were initially deconvoluted using the CASA XPS program and the curve fitting was obtained using OriginPro 2017 version. The Transmission electron microscope (TEM) and high-resolution transmission electron microscope (HR-TEM), as well as EDS spectra and elemental mapping, were obtained using a Hitachi HF-3300 attached to a dual beam-focused ion beam Hitachi NB-5000. Thermis Z with NTIS number NFEC-2019-05-255960 was used to record ultra-high resolution TEM (UHR-TEM), and the corresponding energy dispersive X-ray spectroscopy (EDS) elemental mapping images. X-ray absorption near edge structure (XANES) and Extended X-ray absorption fine structure (EXAFS) at the Pt *L*_3_-edge and Zn K edges were performed at the synchrotron center in Korea; Pohang Accelerator Laboratory with beamline number 8C. The electron-storage ring operates in a voltage range of 4.0 ~ 22.0 keV using Si (111) mode. All the XAS data were recorded at room temperature using the transmittance mode. The XANES and EXAFS spectra were fitted using the Athena and Artemis software packages. The wavelet transform spectra were obtained using the JWT-EXAFS program.^[2]^

**Electrochemical measurements.**

All electrochemical measurements were conducted in a three-electrode cell using the rotating disk electrode (RDE) connected to an electrochemical analyzer (Biologic VMP3) at room temperature. Glassy carbon was used as the working electrode with a diameter of 3 mm. The glassy carbon was coated with the as-synthesized catalyst or commercial Pt/C in all measurements. In short, the catalyst ink was prepared by dispersing 2.5 mg of catalyst powders in 4 mL of deionized water (D.I water), 0.95 mL of isopropanol (IPA), and 0.5 mL of 5 wt % Nafion solution. The resulting solution was sonicated for 1 hour to form a uniform slurry. Subsequently, 3.5 µL of the slurry was drop cast over the working electrode and dried under ambient conditions. Pt wire was used as the counter electrode and Ag/AgCl with saturated KCl was used as a reference electrode to complete the three-electrode cell. All measurements were conducted in a 0.1 M HClO_4_ solution. Oxygen reduction reaction (ORR) measurement was achieved by saturating the 0.1 M HClO_4_ solution with oxygen at a scan rate of 10 mVs^-1^ and a working electrode rotation speed of 1600 rpm. The accelerated degradation tests (ADT) were performed at room temperature in oxygen-saturated 0.1M HClO_4_ by applying cyclic potential sweeps between 0.6 and 0.95 V versus reversible hydrogen electrode (RHE) at a sweep rate of 50 mVs^-1^ for 10k, 20k, and 30k cycles, after which LSV curves were measured for each successive cycle. The electrochemical active surface area (ECSA) measurements were determined by integrating the hydrogen desorption charge on the cyclic voltammetry (CV) at room temperature in nitrogen-saturated 0.1 M HClO_4_. The ECSA is calculated using the following equation; $ECSAPtcat\left( \frac{m^{2}}{g_{Pt}} \right)=\left[ \frac{Q_{H-desorption}(C)}{210\mu C/{cm}^{2}L_{pt}\left( \frac{{mg}_{pt}}{{cm}^{2}} \right)A_{g}} \right] X {10}^{5}$ ( eq1)

Where Q_H-adsorption_ is the hydrogen desorption charge obtained by integrating the area in the CVs. 210 is the charge of a monolayer. Lpt represents the catalyst loading on the electrode surface. A_g_ is the geometric surface area of the glassy carbon electrode (i.e. 0.19625 cm^2^). The kinetic currents were obtained by employing the Koutecky-Levich equation: $j_{k}=\frac{j X j_{d}}{j_{d}-j}$, (eq2) where j_k_ represents the kinetic current density, j is the current density obtained from LSV measurements and j_d_ is the diffusion-limited current density. The mass activities (M.A) of the two catalysts were obtained using the equation $MA=\frac{j_{k}}{Lpt}$ (eq3). The Specific activities were calculated from the mass activities by normalizing with the ECSA.

**MEA Preparation and fuel Cell testing**

Catalysts (Pt/C-19.4 wt% and PtCa/C-20.4 wt%) were mixed with 5 wt% Nafion ionomer solution, isopropanol, and D.I water by ultrasonication for 1 hour to form homogeneous ink. The catalyst slurries were then spray-coated on Nafion 211 membrane (Dupont). In all PEMFC performance tests, commercial Pt/C (TKK, 19.4 wt% Pt) was used as the anode catalyst, and the electrode area for both the anode and cathode was maintained at 5 cm^2^. PtCa/C was employed as a cathode catalyst. The cathode catalyst loadings were maintained at 0.10 mg_metal_ cm^-2^ while the anode loading was maintained at 0.05 mg_Pt_ cm^-2^. The MEAs were assembled with commercial gas diffusion layers (GDL, SGL 39 BC) without hot-pressing. Membrane electrode assemblies (MEAs) were prepared by using catalyst-coated membranes (CCMs). The cell was operated at 80 ^0^C with a back pressure of 0.5 bar. Pure hydrogen and oxygen with 100% relative humidity (RH) were supplied to the anode and cathode at flow rates of 300 mL min^-1^ and 1000 mL min^-1^ respectively. Fuel cell polarization curves were recorded using a PEMFC test station (Scitech Inc., KOREA) with an electronic load (PLZ664 WA, Kikusui). The accelerated durability test (ADT) was conducted by applying cyclic sweeps from 0.6 V to 0.95 V with a rise time of 0.5 s according to US DOE protocol.^[3]^ Polarization curves and electrochemical impedance spectroscopy (EIS) (@100 mA cm^-2^) were recorded at the start and after 10k, 20k, and 30k cycles of the durability testing protocol. In the impedance circuit, CdLA and CdLC represent the double layer capacitance of the anode and the cathode, respectively, RΩ represents the internal resistance of the membrane, Rf,A and Rf,C for the charge transfer resistance of the anode and cathode, and Zw for the Warburg impedance associated with mass transport. All polarization curves indicated are without iR correction.

**Computational details**

To investigate the oxygen reduction reaction (ORR) over Pt and Pt-Ca model systems, we construct a three-layer slab model cut along the (111) direction. Our slab models consist of a (3x3) unit cell with a total of 27 Pt atoms and 9 Pt surface sites, as discussed further below. Spin-polarized electronic structure calculations were performed within the density functional theory^[4,5]^ framework using the VASP ab-initio package.^[6–8]^

The generalized gradient approximation (GGA) was utilized, specifically the Perdew-Burke-Ernzerhof (PBE) functional^[9]^ using the Vienna Ab initio Simulation Package (VASP) with the VASPsol extension.^[6,8,10,11]^ To account for dispersion effects, the D4 correction method, recently developed by Grimme and colleagues, was applied.^[12,13]^ Numerical integration in the reciprocal space was conducted using a 4×4×1 k-point mesh, and the valence electron density was represented through a plane-wave basis set with a kinetic energy cutoff of 550 eV. Both the k-point grid and energy cutoff were carefully converged to achieve a total energy accuracy within 1 meV/atom. Structural optimization was considered converged when the maximum residual forces on atoms in the supercell fell below 0.03 eV/Å. The total energy in the self-consistent field cycle was converged to a threshold of 10^−6^ eV. Geometry optimizations were performed using the conjugate gradient method. To avoid interactions between periodic images, a vacuum layer of at least 14 Å was introduced between slabs. Details involving the theoretical studies is added in Notes 1 to 6 of this supplementary file.


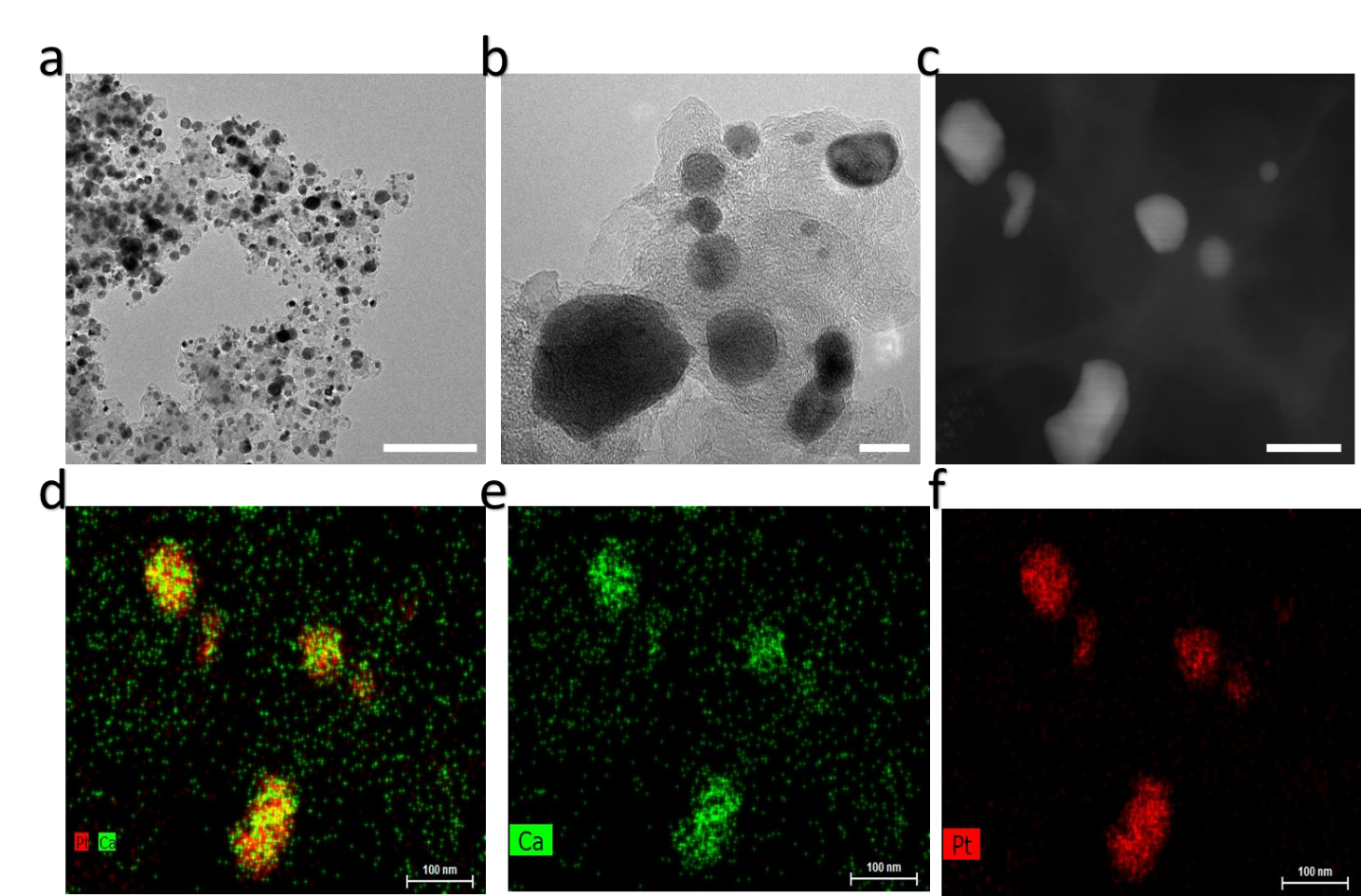


**Figure S1.** a and b) TEM images of PtCa/VC annealed at 850 ^o^C on Vulcan carbon (VC) as support. Scale bars for a and b are 100 and 10 nm respectively. c) HAADF-STEM image of selected PtCa NPs and d, e, and f) corresponding EDS elemental mappings (Scale bars are in 100 nm).


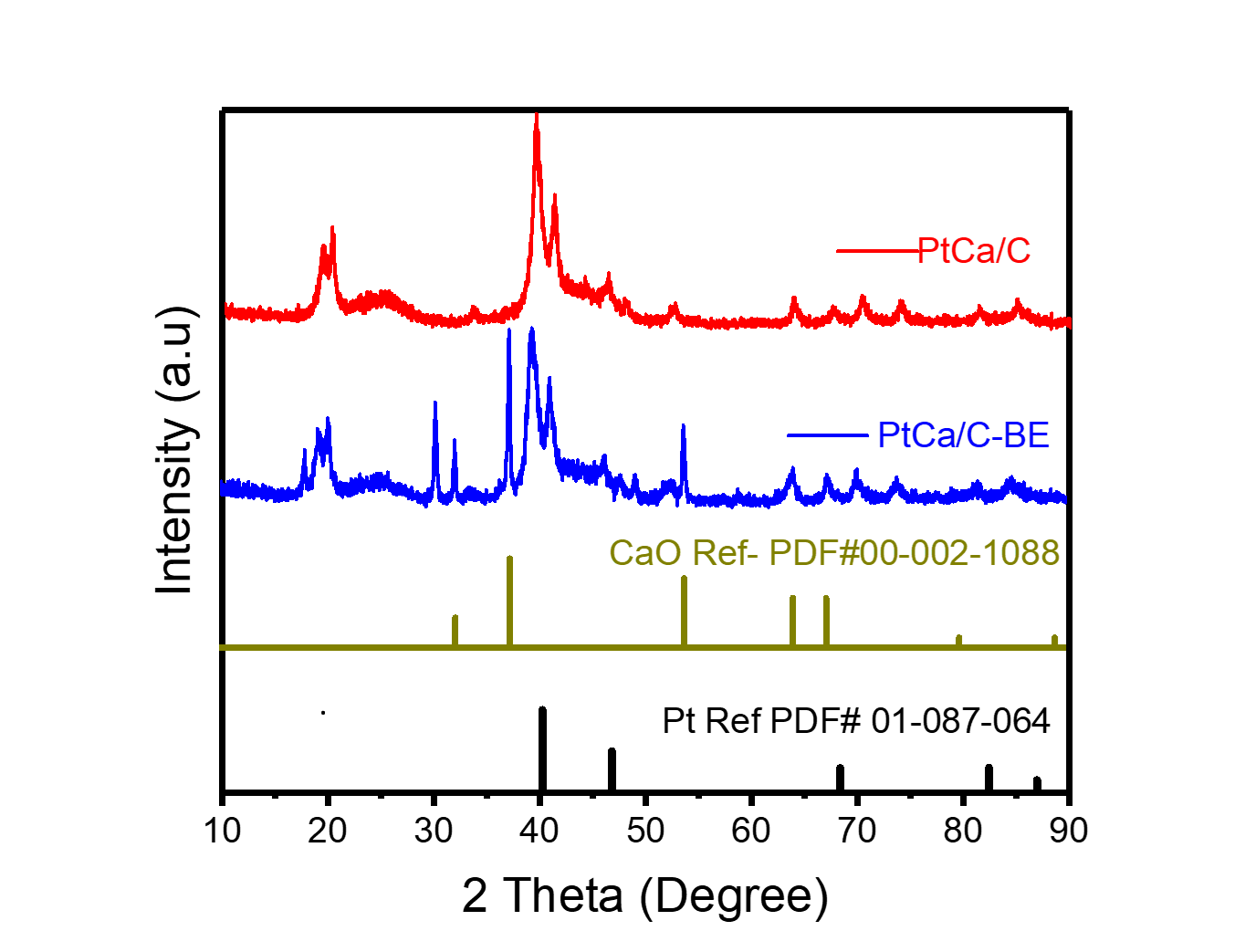


**Figure S2.** XRD patterns of the PtCa catalyst obtained after 850 ^o^C annealing treatment. The blue and red XRD patterns represent the sample before (BE) and after acid treatment respectively.


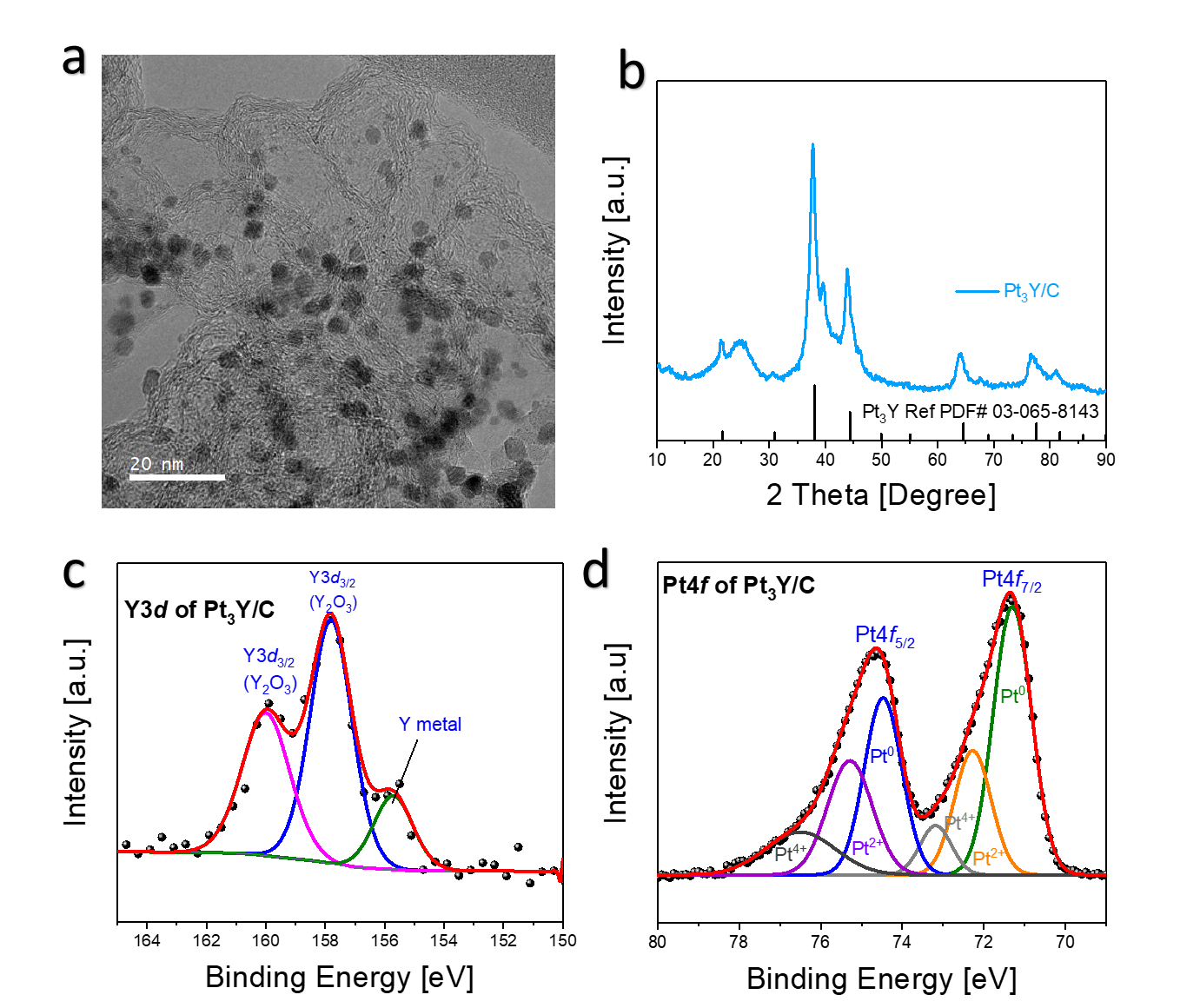


**Figure S3**. a) TEM image, b) XRD patterns, and high resolution XPS spectra of c) Y 3*d* and d) Pt 4*f* scan of Pt_3_Y/C.


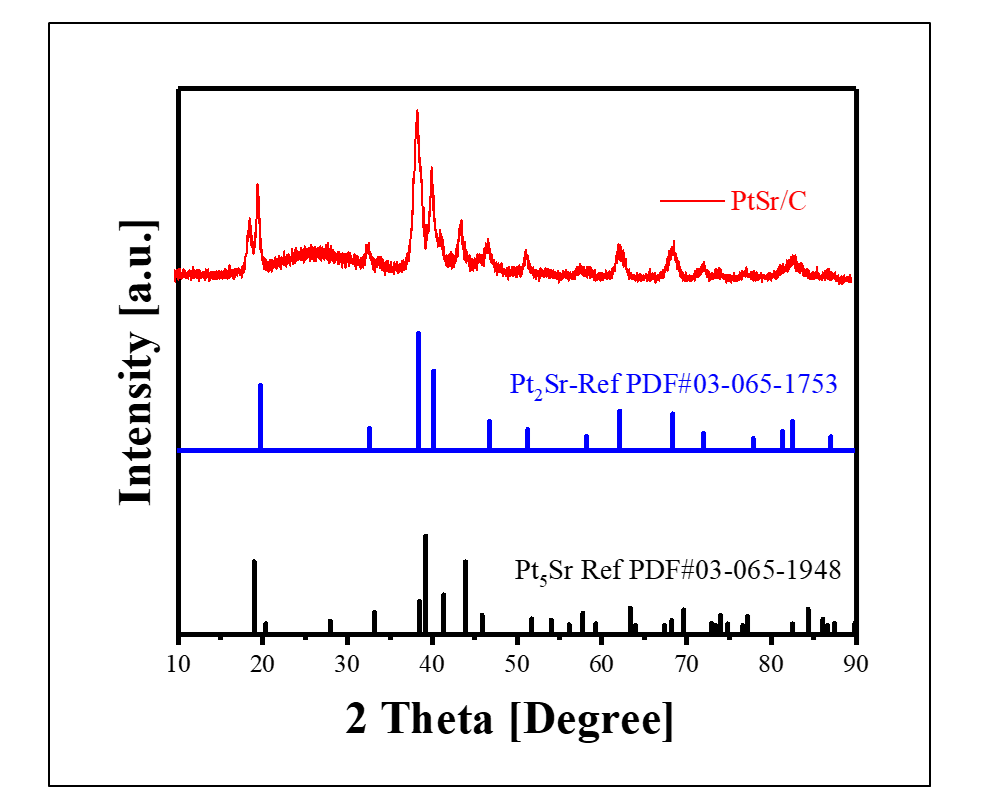


**Figure S4.** XRD patterns of PtSr/C synthesized at 900 ^o^C.


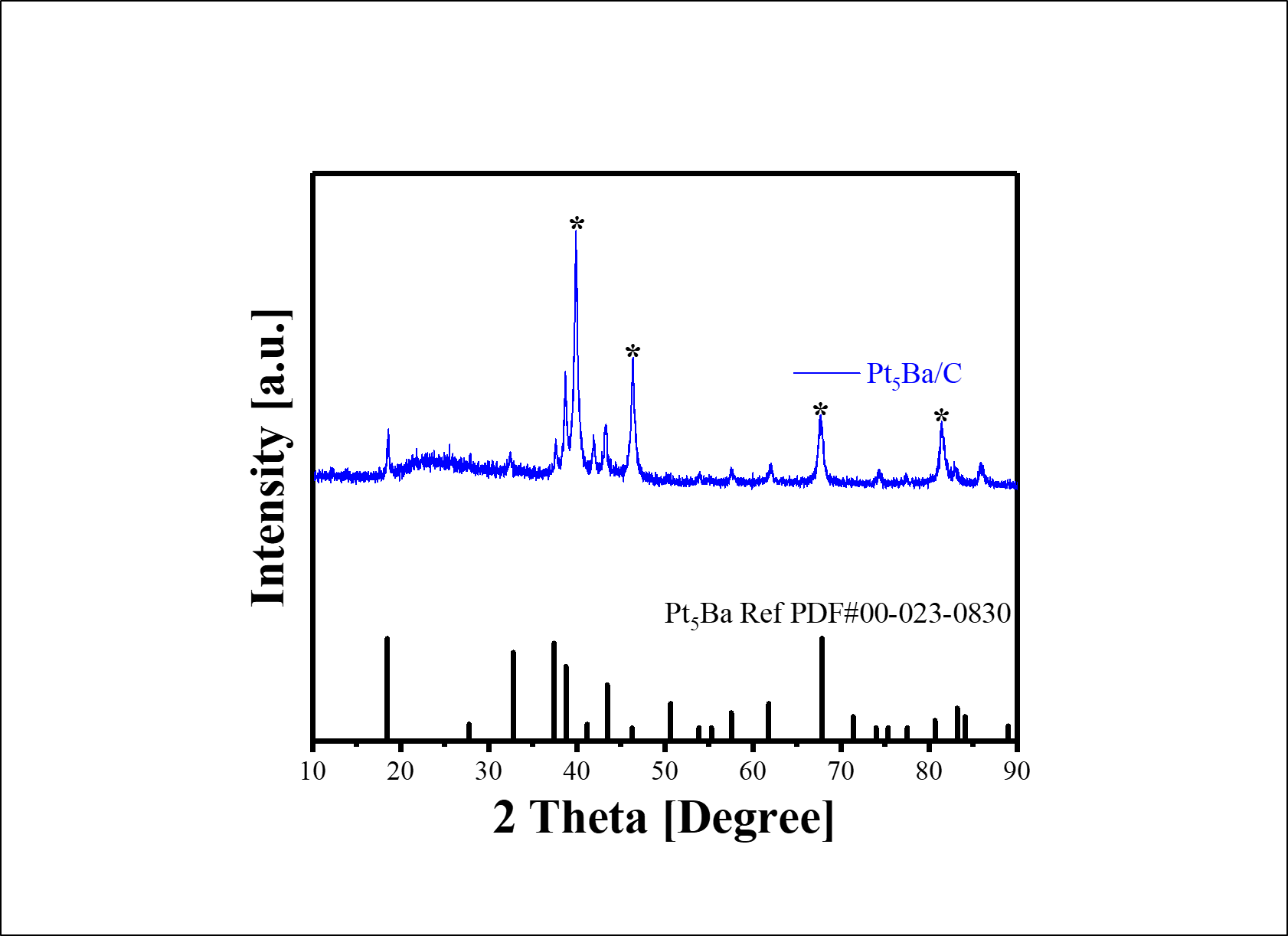


**Figure S5**. XRD patterns of PtBa/C synthesized at 900 ^o^C. Peaks with * corresponding to pure Pt diffraction peaks.

**
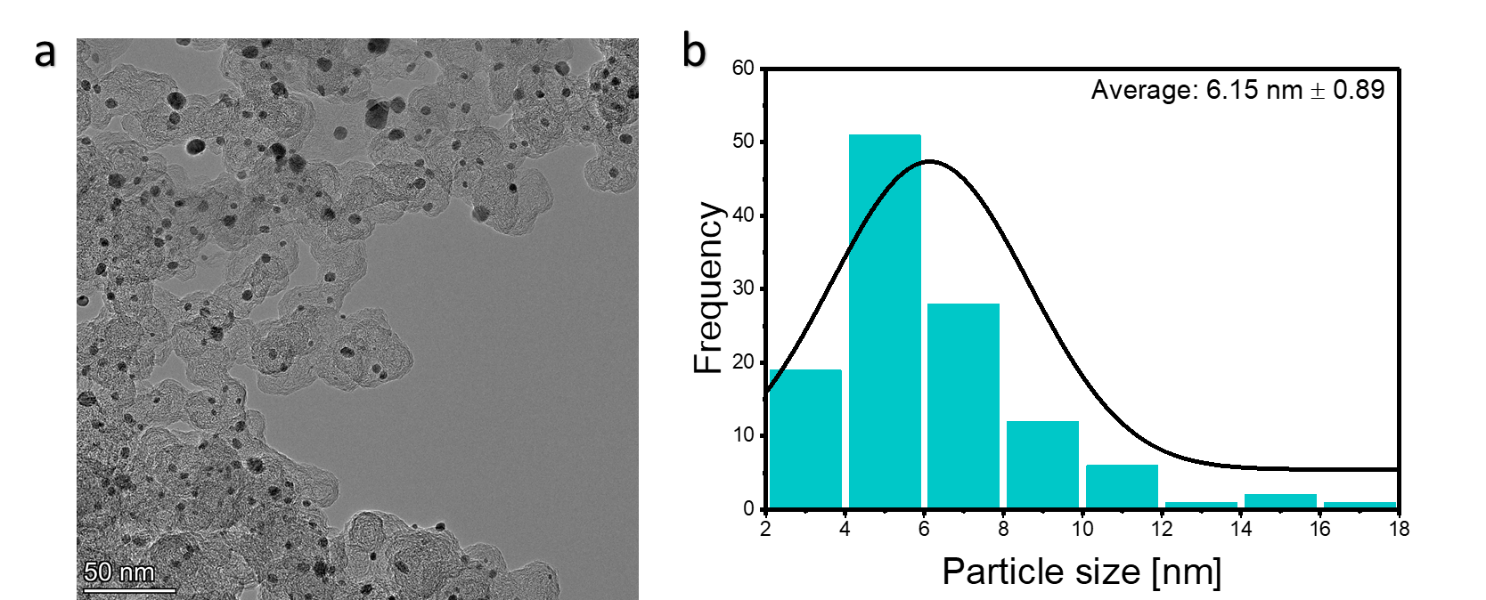
**

**Figure S6**. a) TEM image of PtCa/C and b) histogram of the corresponding particle size distribution.


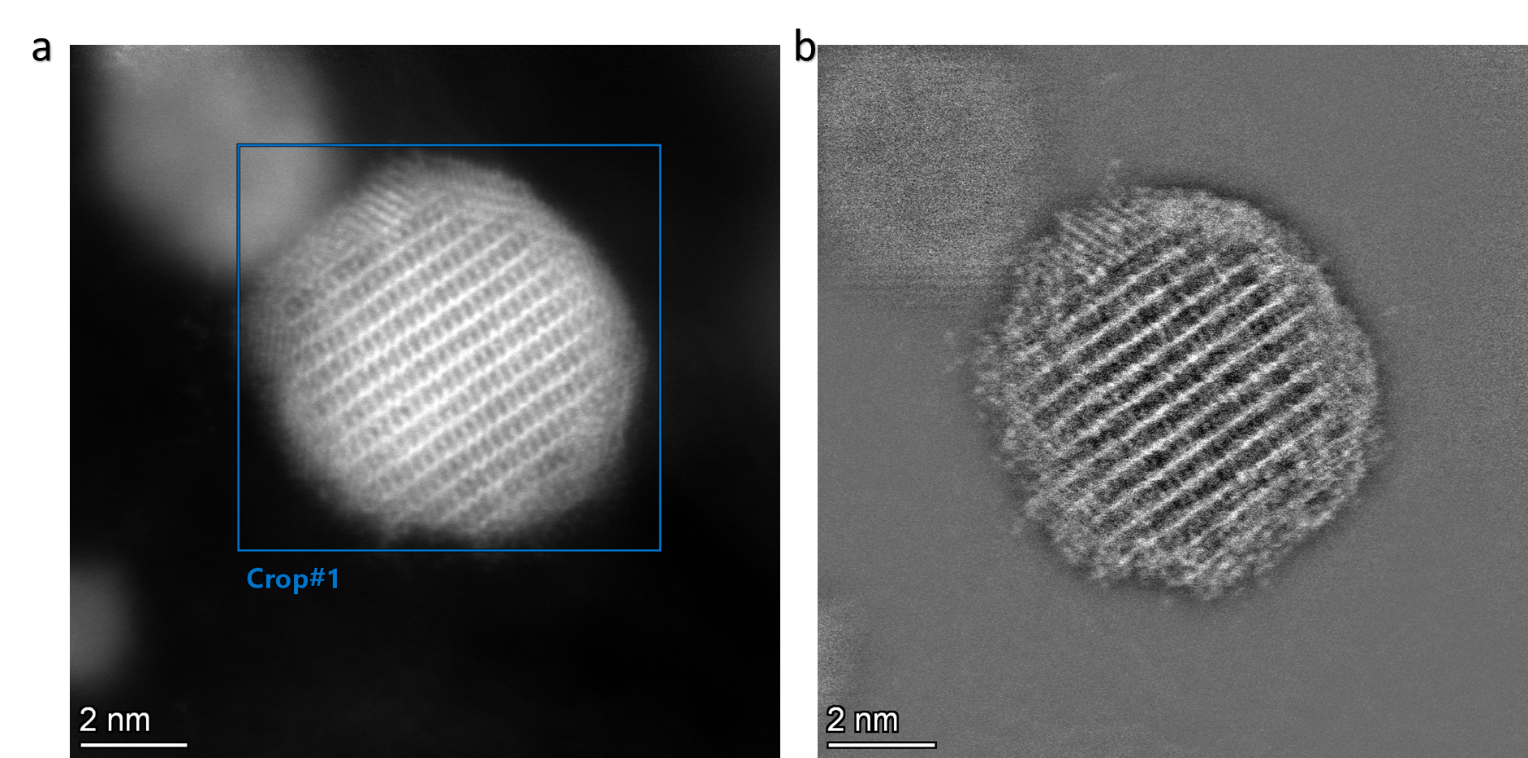


**Figure S7.** a) HAADF-STEM image of the PtCa alloy annealed and acid-etched after the 850 ^o^C annealing treatment and b) the corresponding gray-scale image showing the Intermetallic core, Pt-rich shell arrangement.


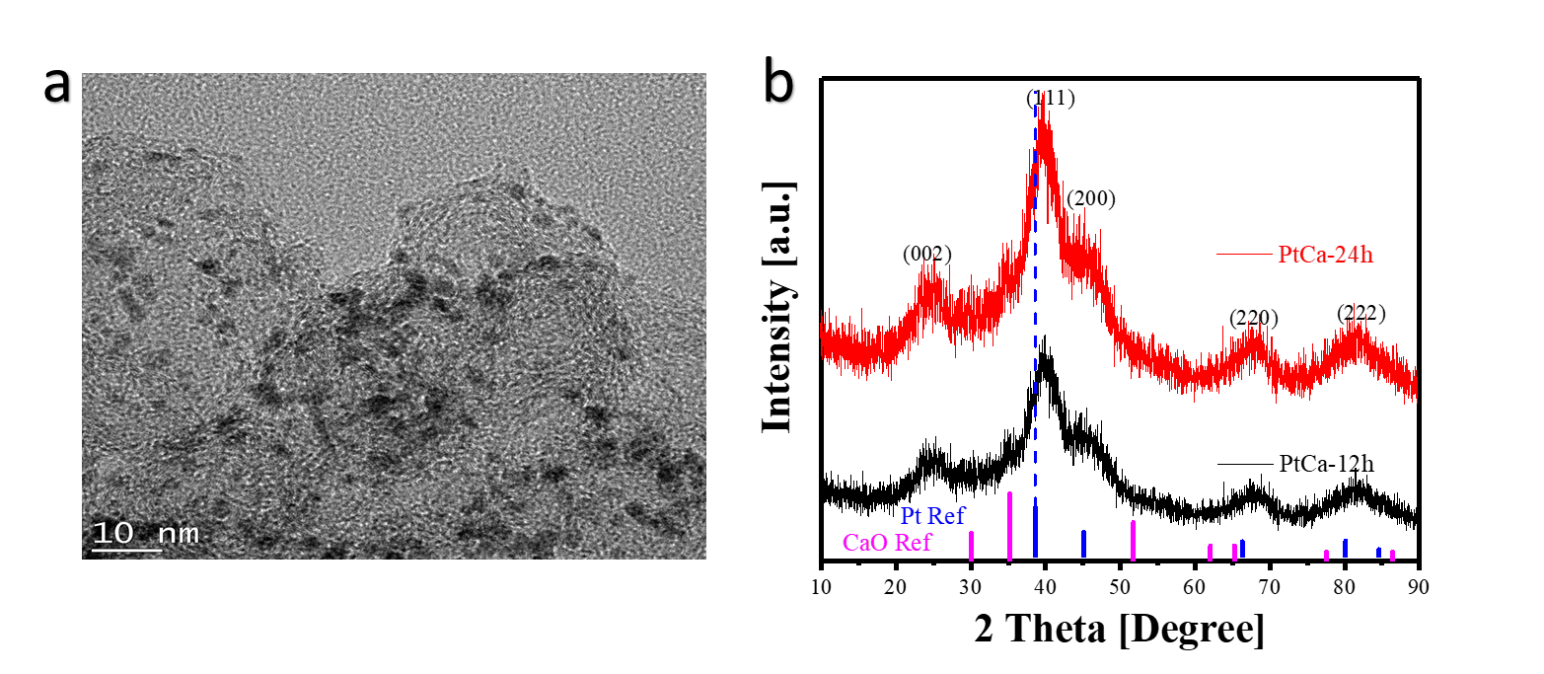


**Figure S8**. a) TEM image of PtCa/C as prepared by heating at 165 ^o^C for 24 h before annealing at high temperature (850 ^o^C). b) XRD patterns of as-prepared catalyst for 12 h (black) and 24 h (red) of reaction.


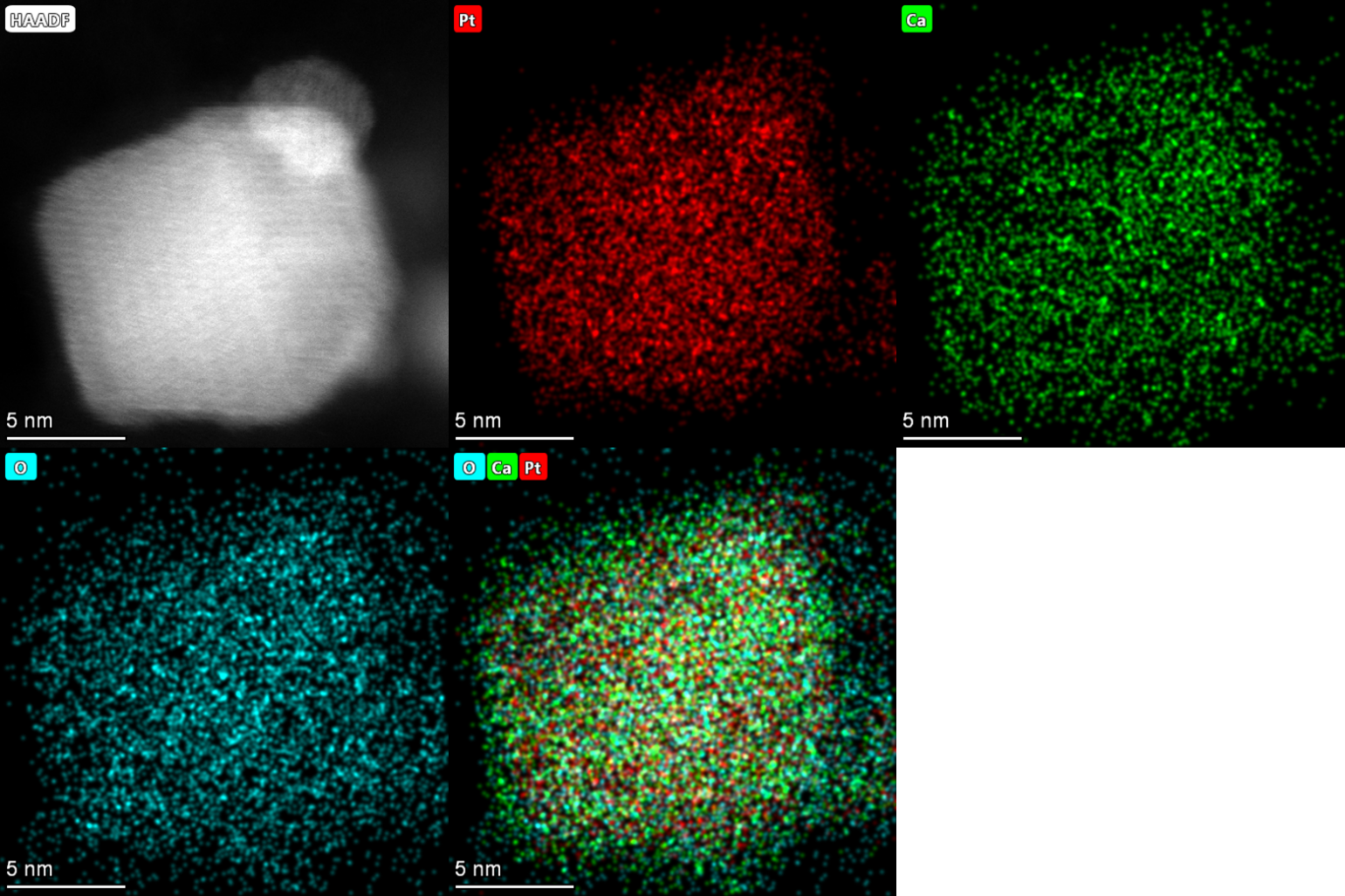


**Figure S9**. HAADF-STEM image and the corresponding EDS elemental mapping images of PtCa/C prepared at 850 ^o^C before acid etching. The area corresponding to Ca (green) and O (turquoise blue) is larger than that of Pt (red), where Pt is mainly centered in the core of the particle, evincing the presence of CaO protection.


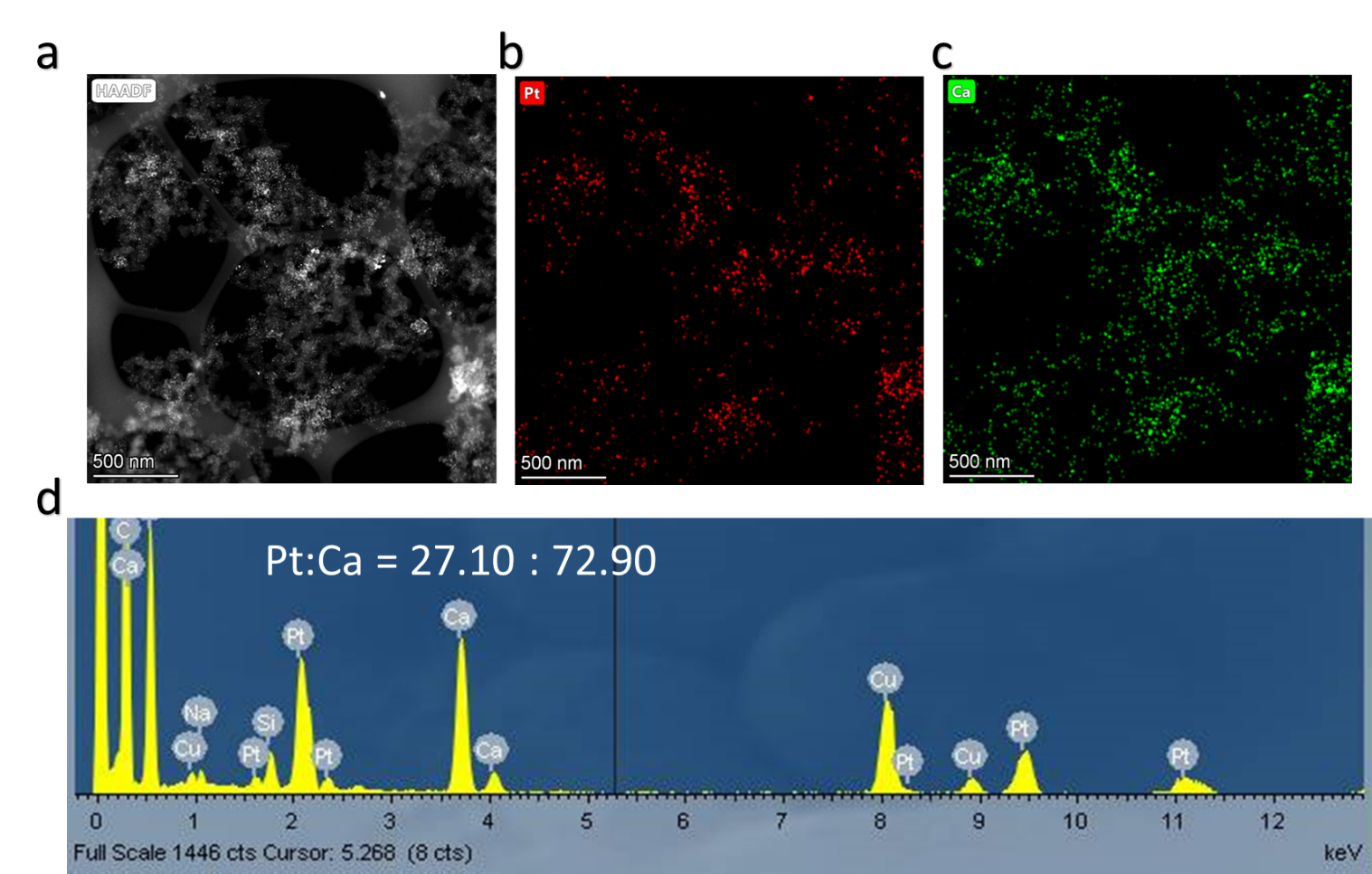


**Figure S10**. a) STEM image and the corresponding EDS mappings for b) Pt c) Ca and d) EDS survey spectrum and elemental composition of as-prepared PtCa catalyst treated at 165 ^o^C for 24 h.

**
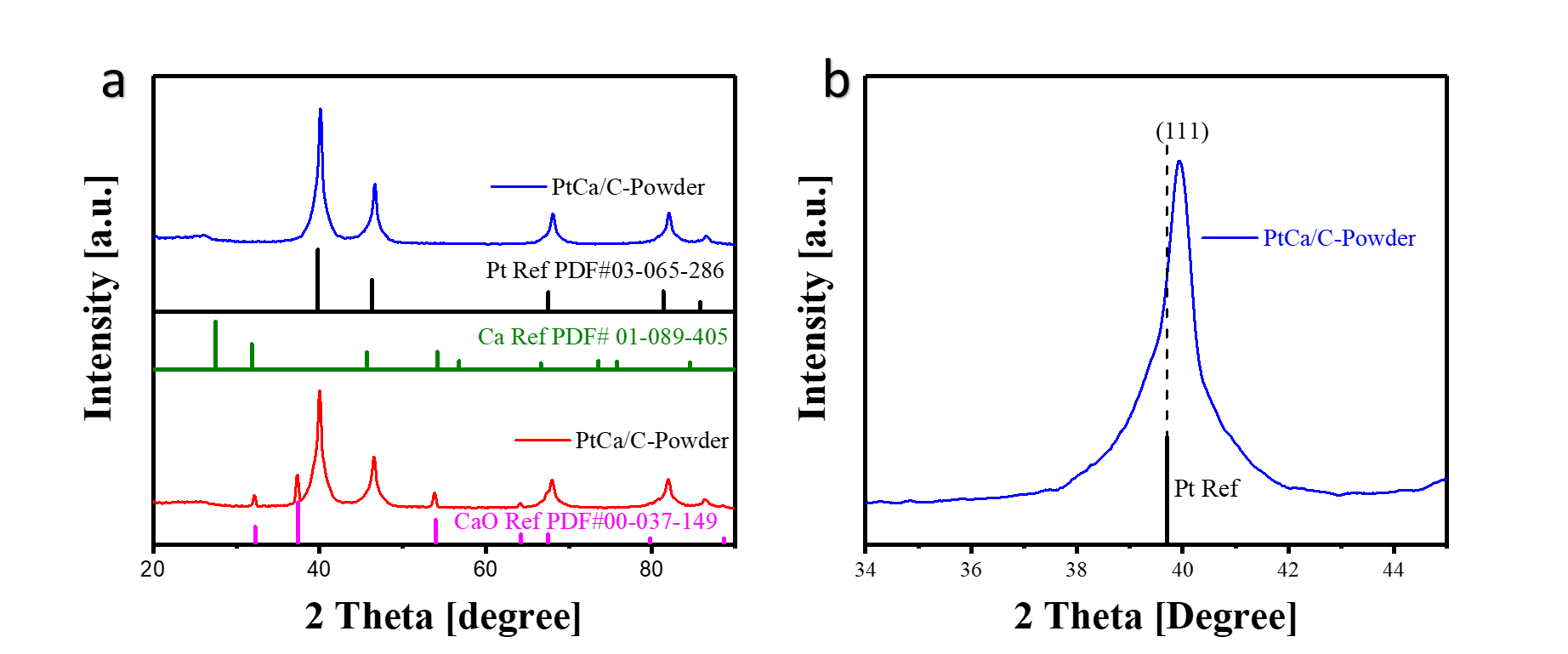
**

**Figure S11**. a) XRD patterns for PtCa/C-Powder synthesized at 850 ^o^C using PtxMg approach before(red) and after acid etching (blue). b) Pt (111) XRD peak shift for PtCa/C-Powder.

**
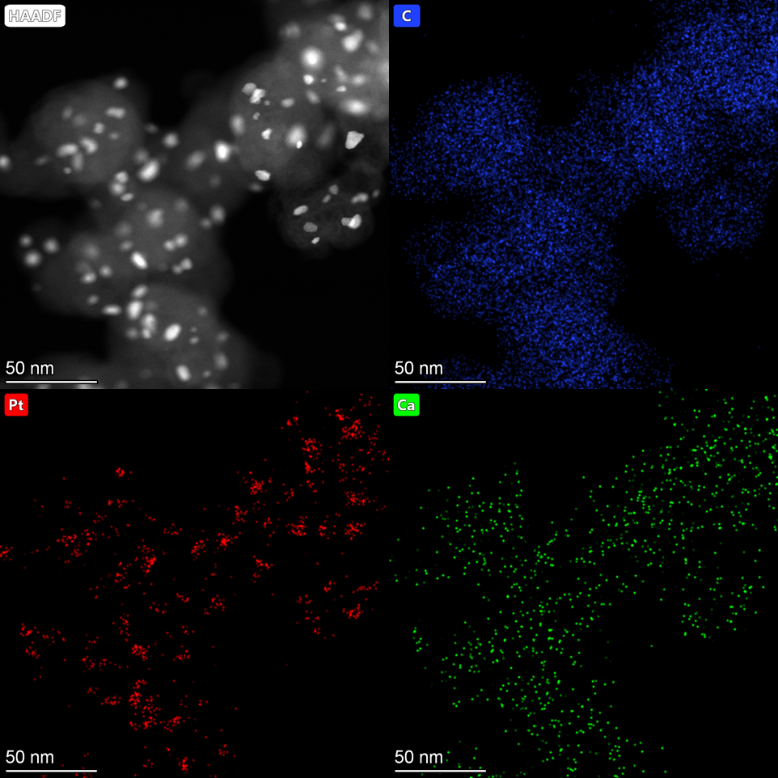
**

**Figure S12**. Large area STEM image and corresponding EDS elemental mapping of PtCa//C NPs showing C (blue), Pt (red), and Ca (green).


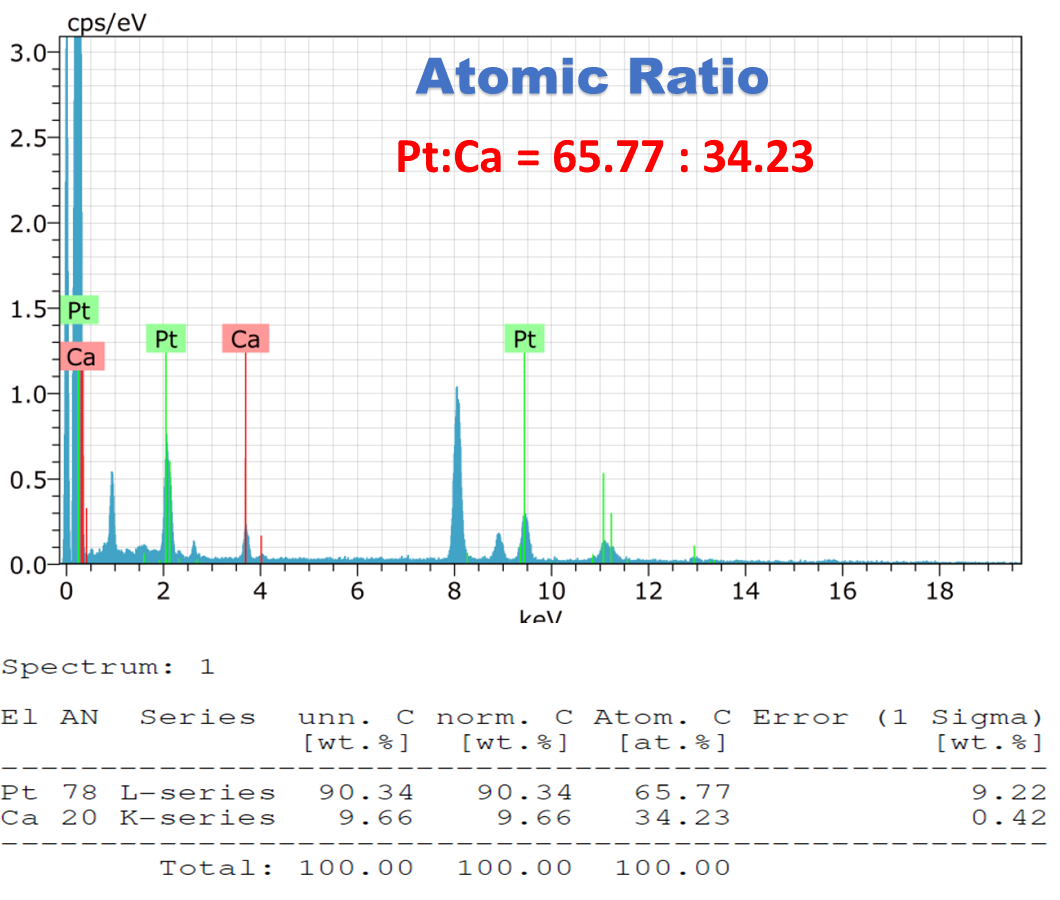


**Figure S13.** EDS Survey spectrum of PtCa/C catalyst annealed at 900 ^o^C with an atomic composition of 65.77% for Pt and 34.23% for Ca.


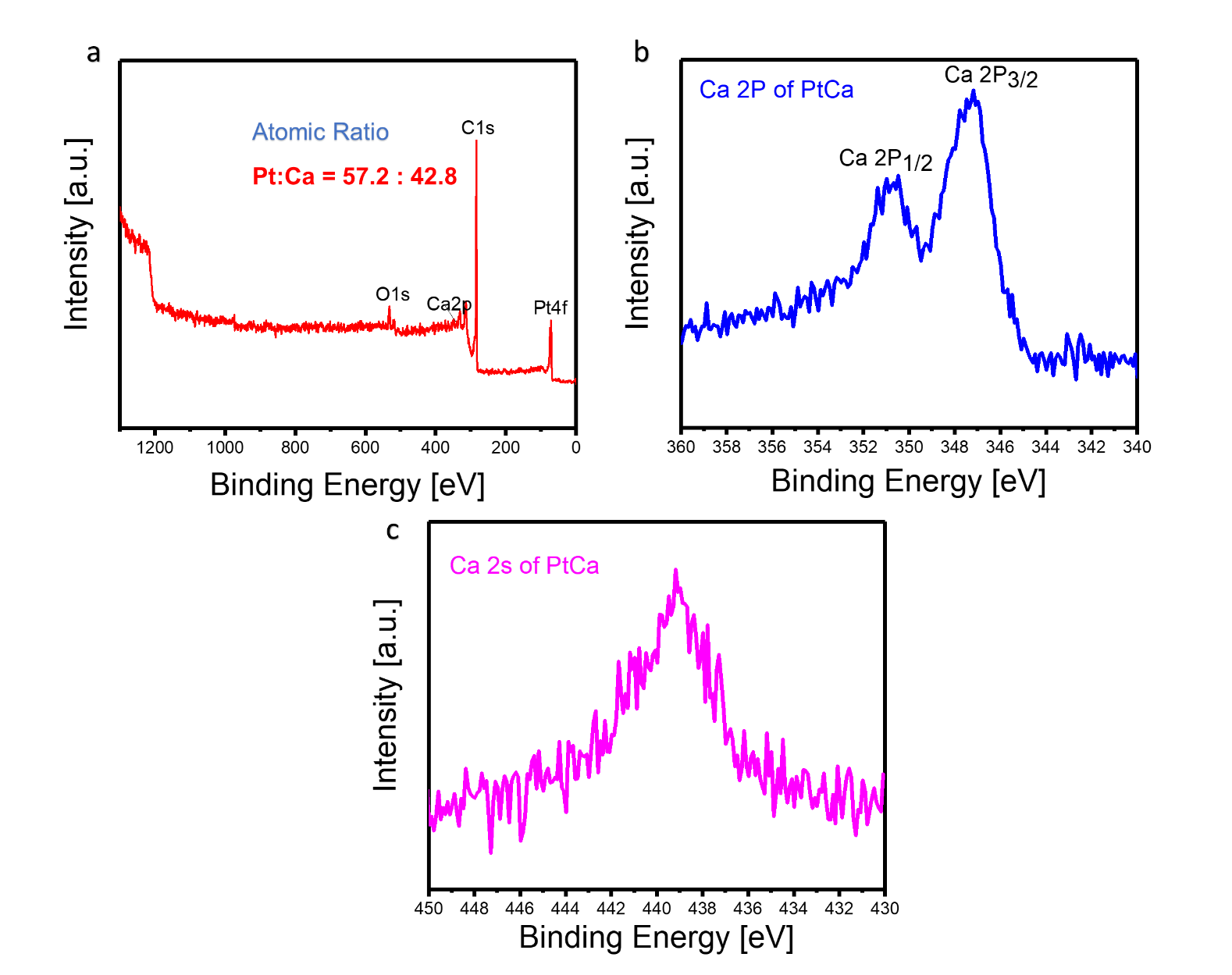


**Figure S14**. a) XPS survey spectrum and high-resolution XPS spectra b) Ca 2*p*, c) Ca 2*s* of the PtCa/C sample.


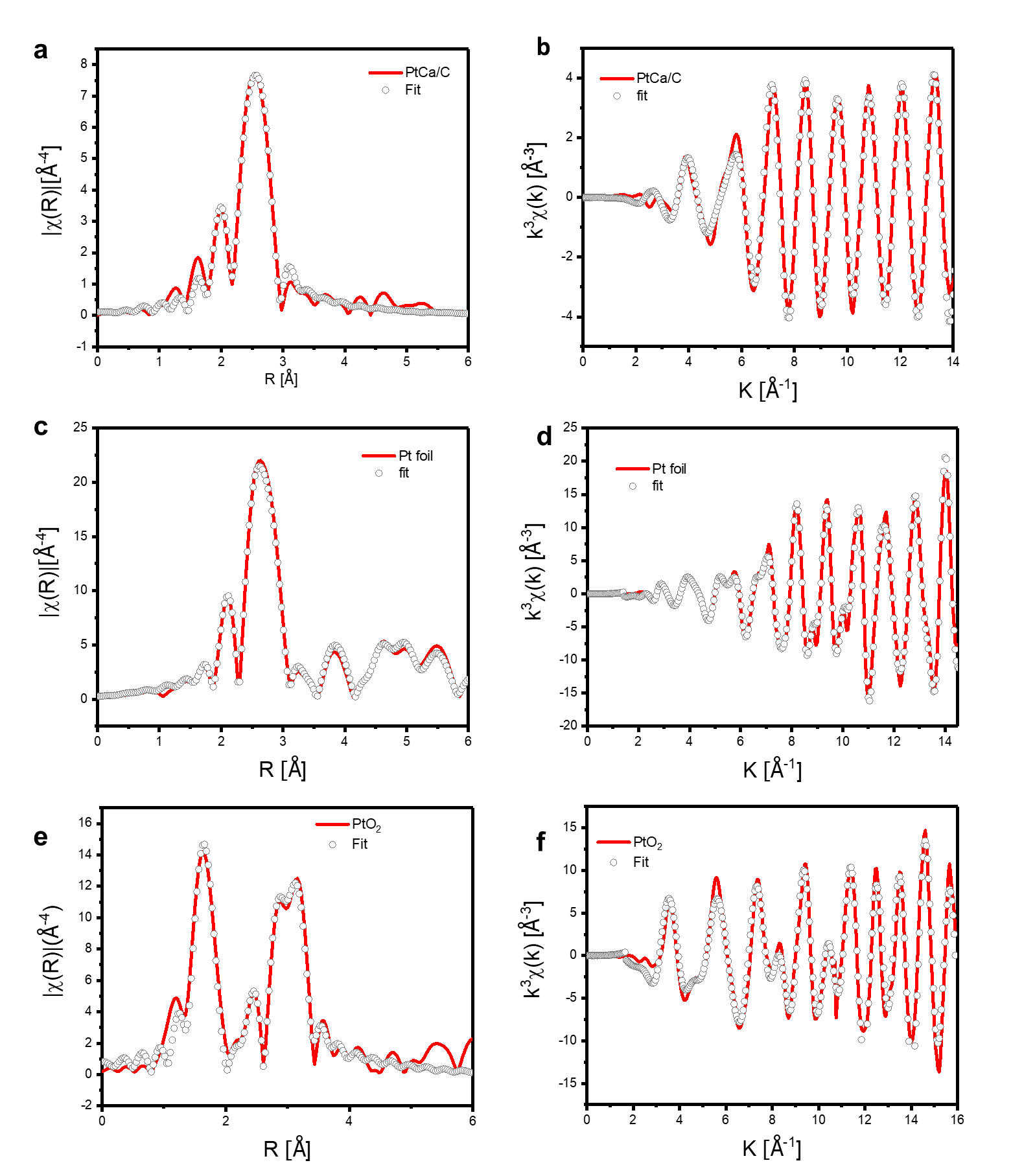


**Figure S15.** Extended X-ray absorption fine structure (EXAFS) analysis. Experimentally obtained data (red line) and the fitting results (white circles) of the Fourier transformed EXAFS spectra of PtCa/C, Pt foil, and PtO_2_ in a, c, and e) R-space and b, d, and f) K-space, respectively.


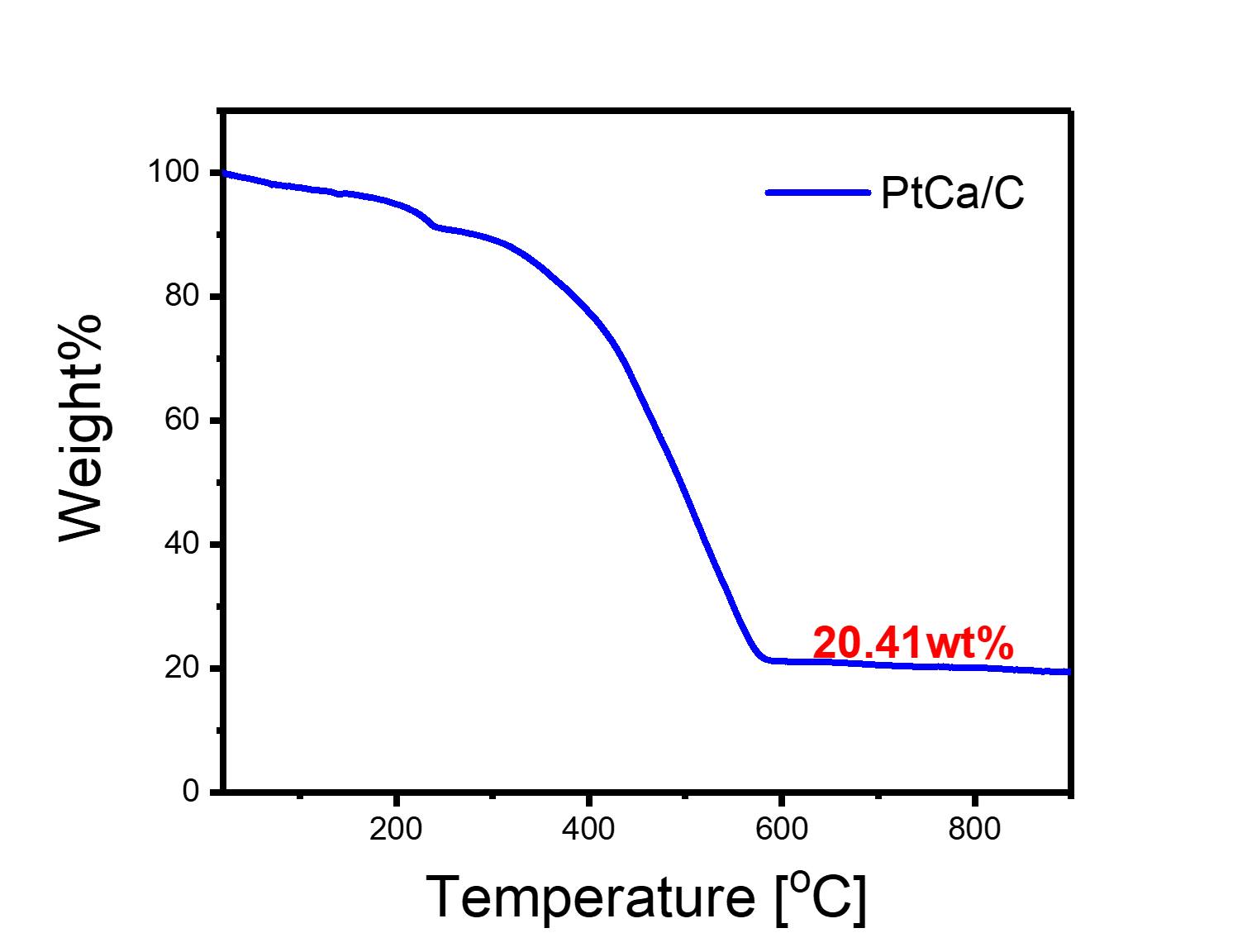


**Figure S16**. TGA data recorded for the PtCa/C.


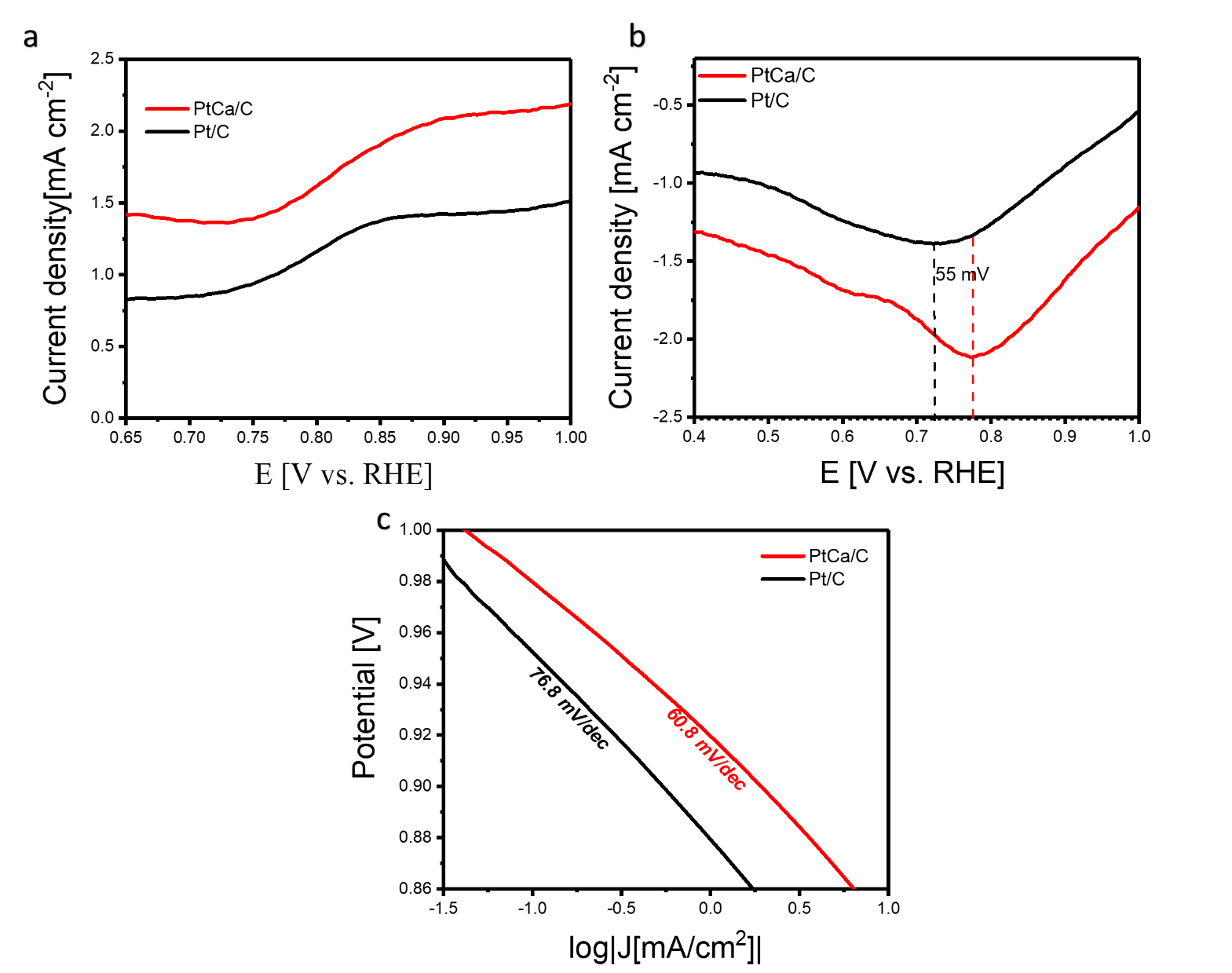


**Figure S17.** a and b) The Enlarged portion of the CV in Figure 3b showing the difference in potential between Pt oxidation and reduction peaks and c) Tafel plots for PtCa/C and Pt/C.


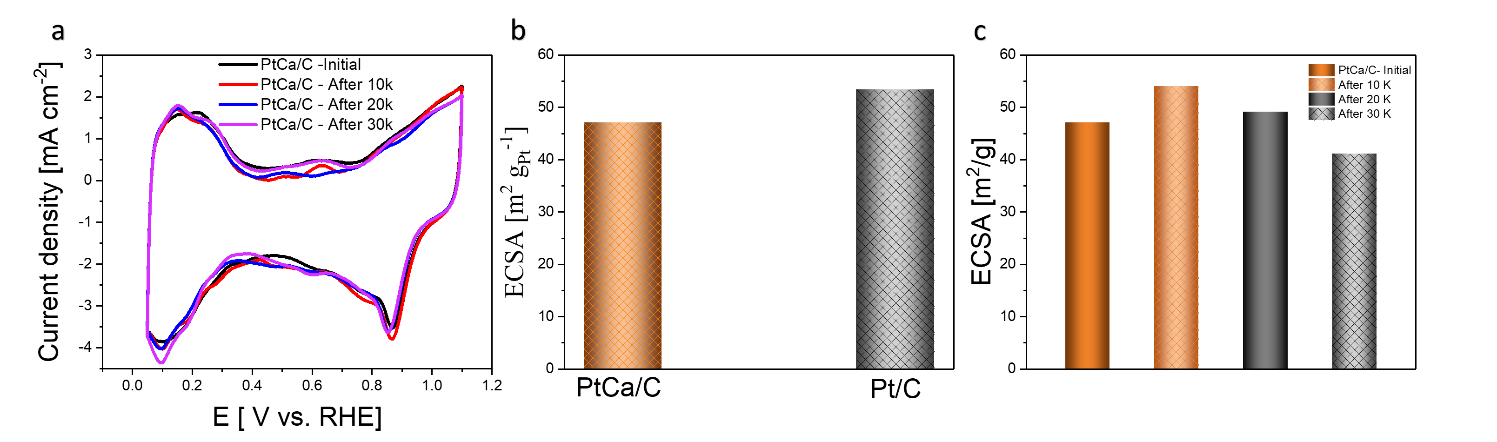


**Figure S18**. a) CV’s recorded in O_2_ saturated 0.1 M HClO_4_ at a scan rate of 50 mV s^-1^ for PtCa/C before and after 10k, 20k, and 30k of ADT test b) histograms of the ECSA at BOL for Pt/C and PtCa/C and c) PtCa at BOL, after 10k, 20k and 30k EOL, respectively.


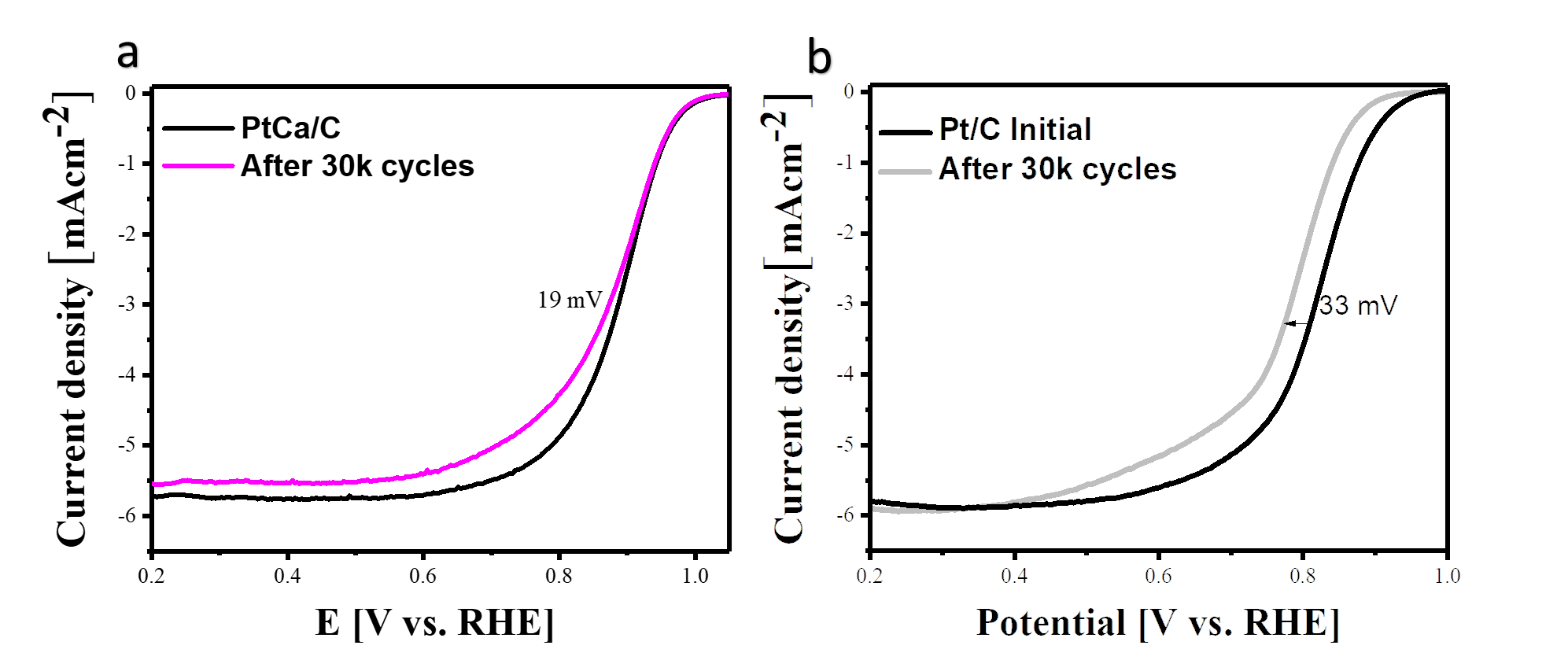


**Figure S19.** Linear sweep voltammetry (LSV) curves of a) PtCa/C and b) Pt/C before and after 30,000 ADT.


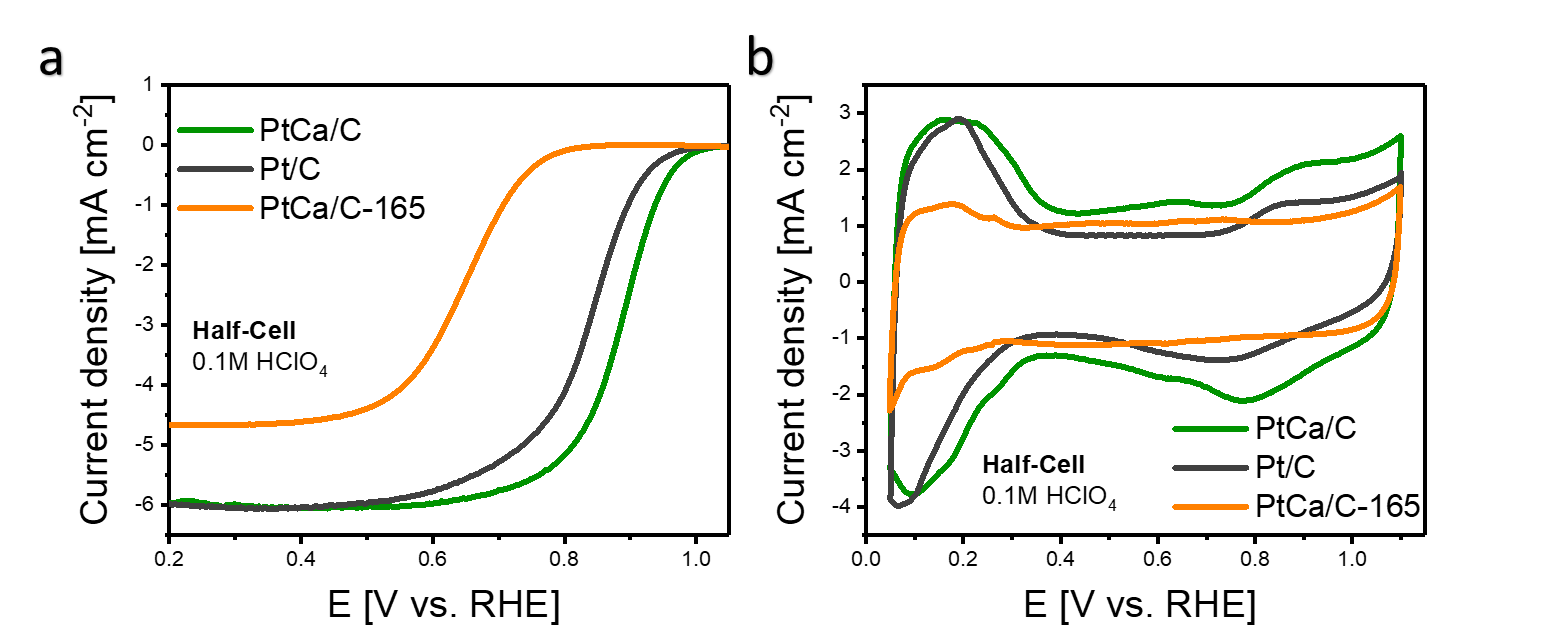


**Figure S20.** a) Linear sweep voltammetry (LSV) curves and b) cyclic voltammograms (CVs)s of PtCa/C-165, PtCa/C and Pt/C. The PtCa/C-165 represents the sample prepared at 165 ^o^C before the high temperature annealing.


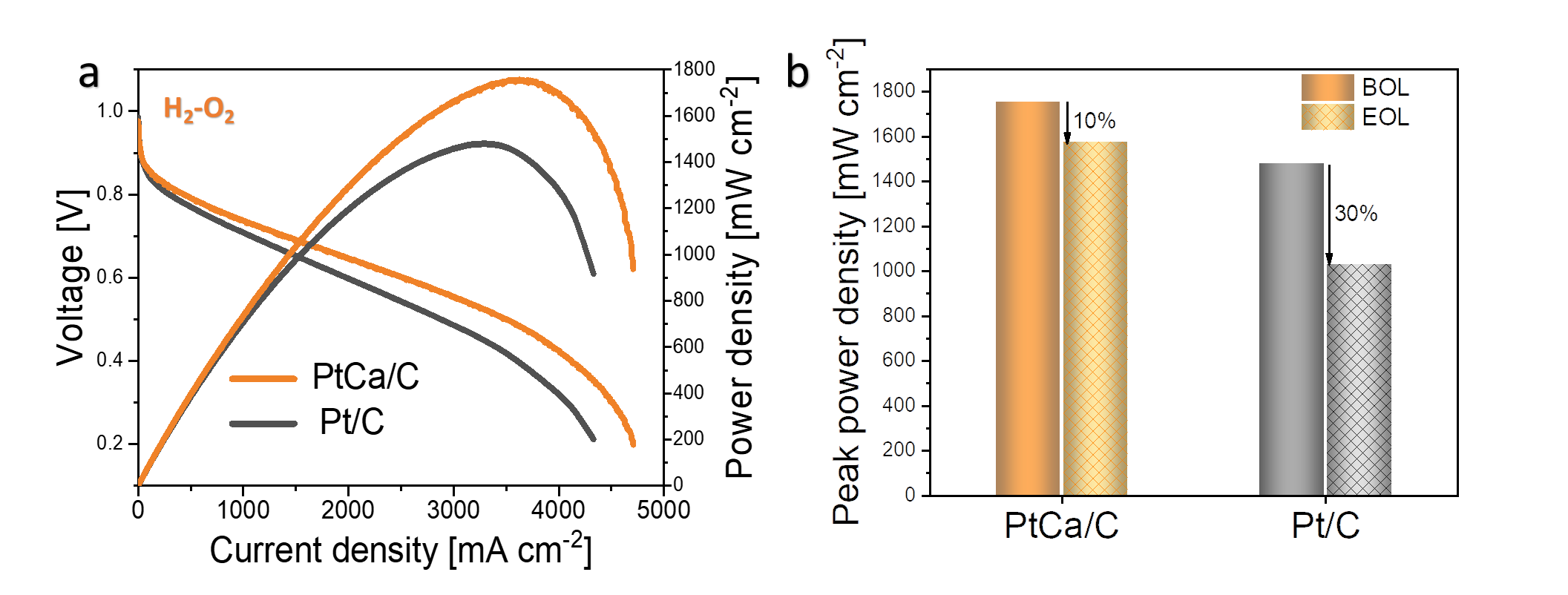


**Figure S21.** a) H_2_-O_2_ fuel cell polarization curves of commercial Pt/C and PtCa/C recorded before ADT at 0.5 bar back pressure and 100% RH. b) The histogram of the peak power densities before and after 30 k ADT recorded under the same conditions.


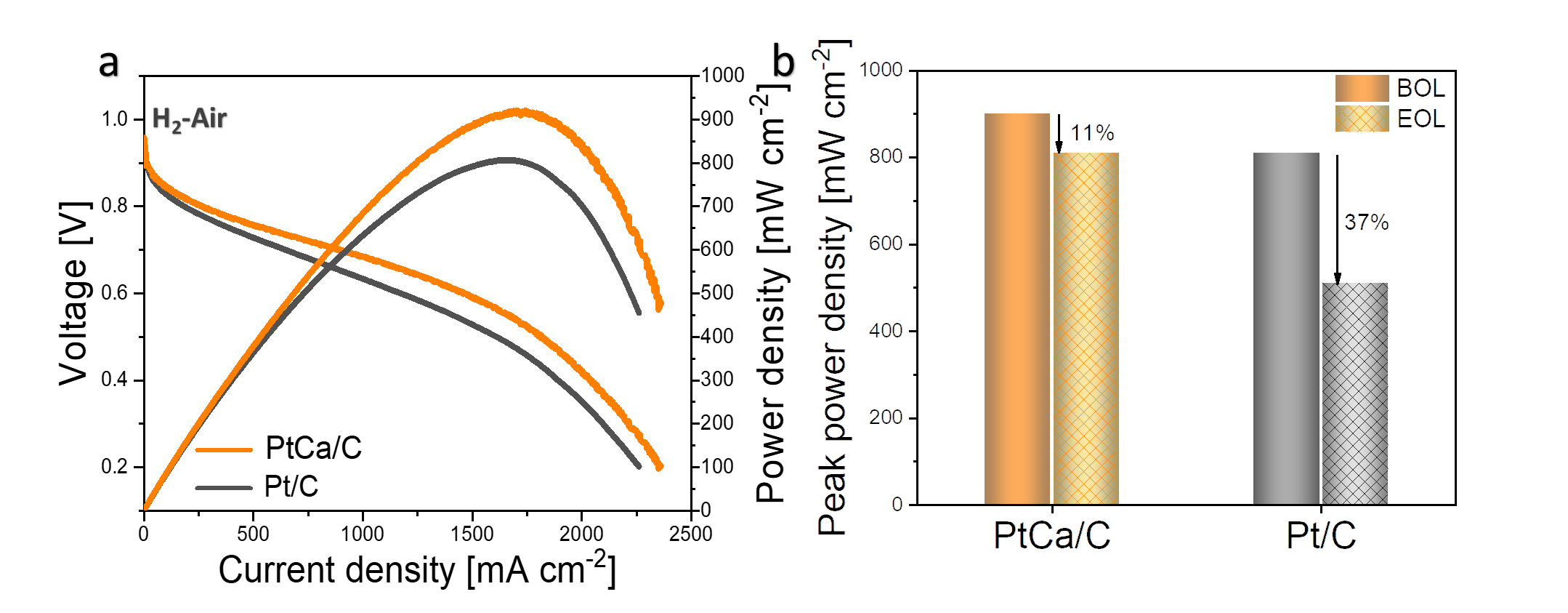


**Figure S22.** a) H_2_-air fuel cell polarization curves of commercial Pt/C and PtCa/C recorded before ADT at 0.5 bar back pressure and 100% RH. b) The histogram of the peak power densities before and after 30 k ADT recorded under the same conditions.


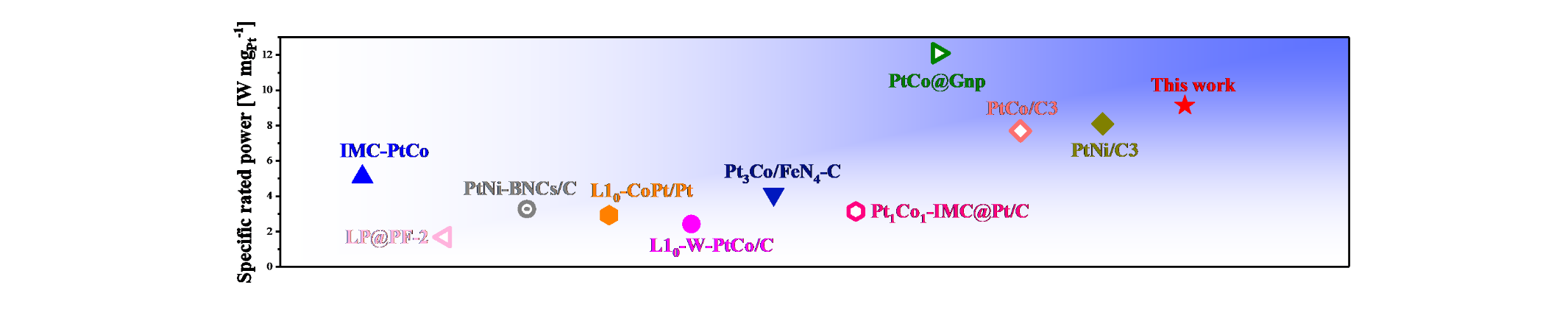


## **Figure S23**. Specific rated power of PtCa/C compared to recently published high-performing PEMFC electrocatalysts.^[14–22]^


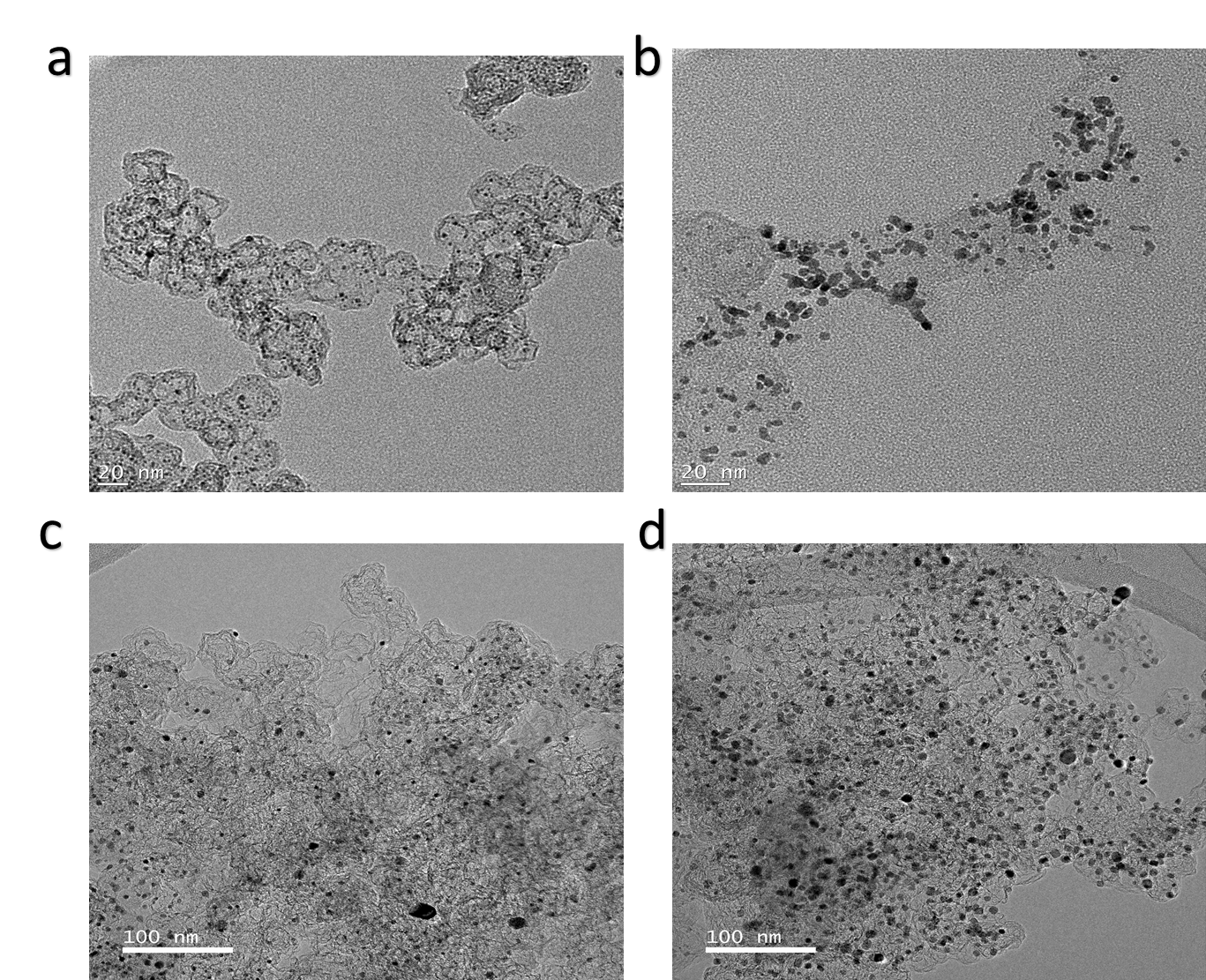


**Figure S24**. TEM images before and after 30k ADT respectively for a and b) commercial Pt/C, c and d) PtCa/C.


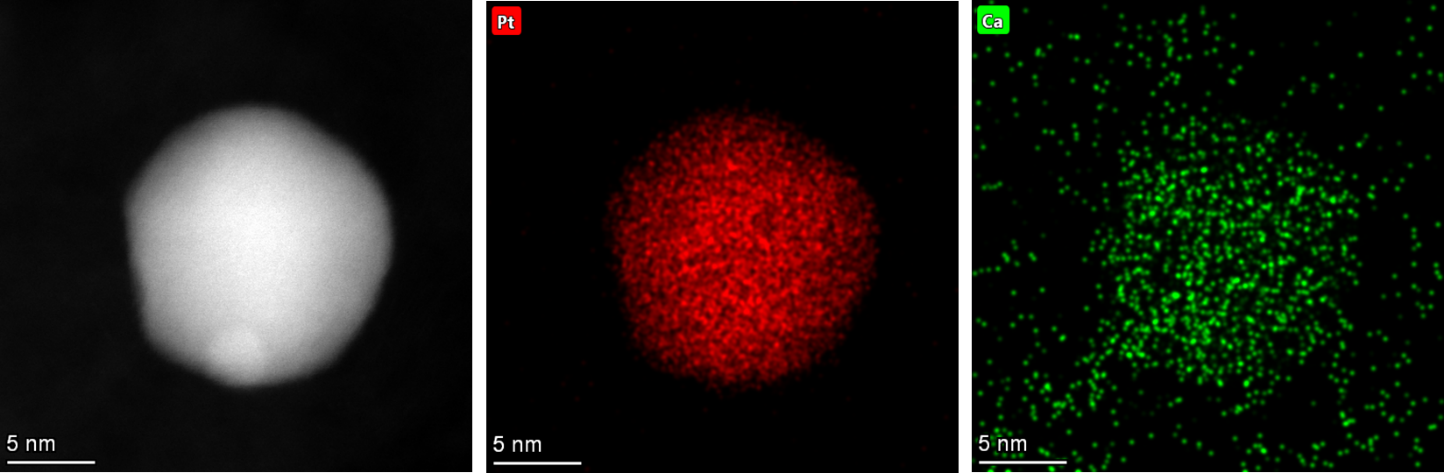


**Figure S25.** HAADF-STEM image and the corresponding EDS of a single PtCa after 30 k ADT.


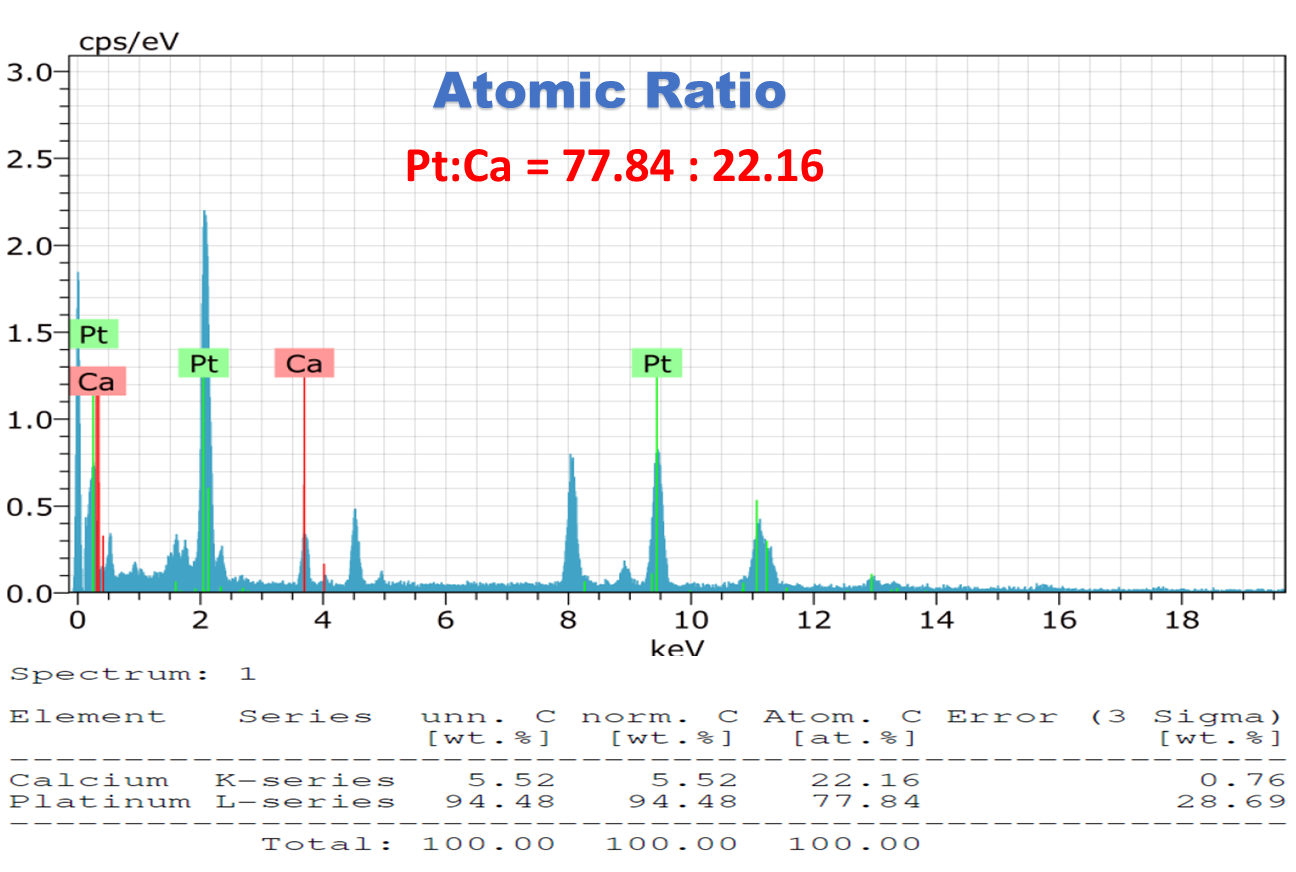


**Figure S26.** EDS spectrum for PtCa/C after 30k ADT test.


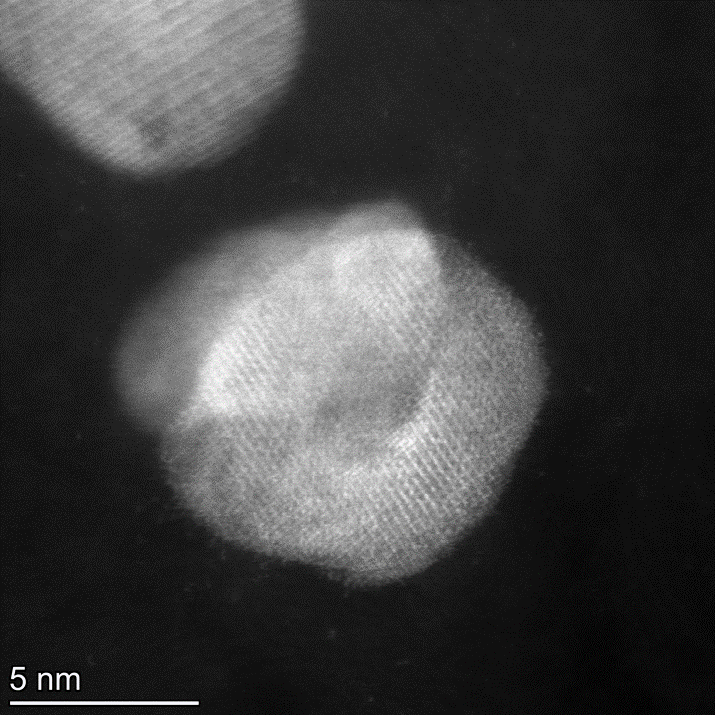


**Figure S27.** HAADF-STEM images of PtCa particles after 30 k ADT.


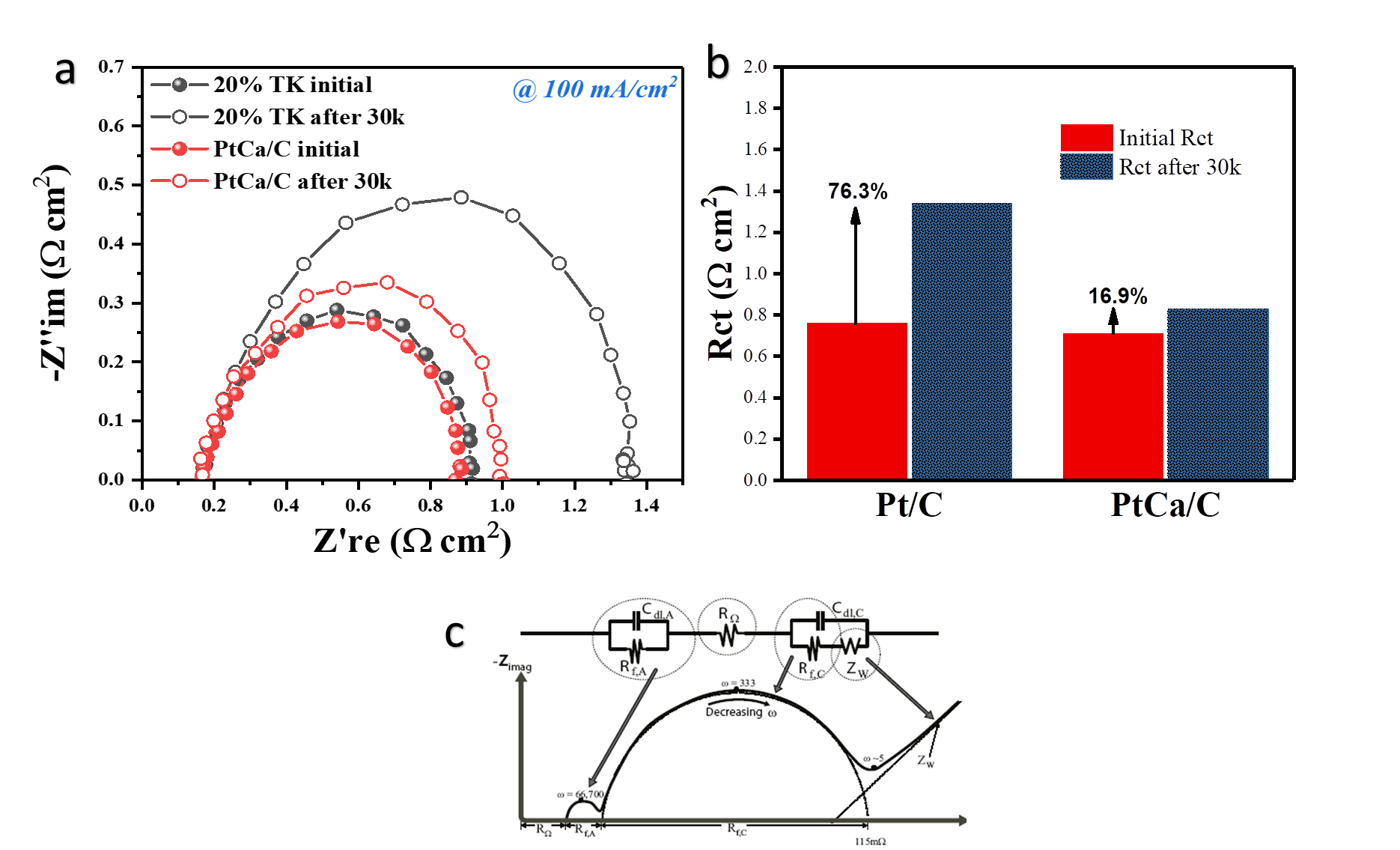


**Figure S28.** a) PEMFC Electrochemical impedance spectra (Nyquist plots) and b) charge transfer resistance (Rct) changes for PtCa/C and Pt/C before and after 30k potential cycling. c) Equivalent circuit employed for the measurement of the impedance.


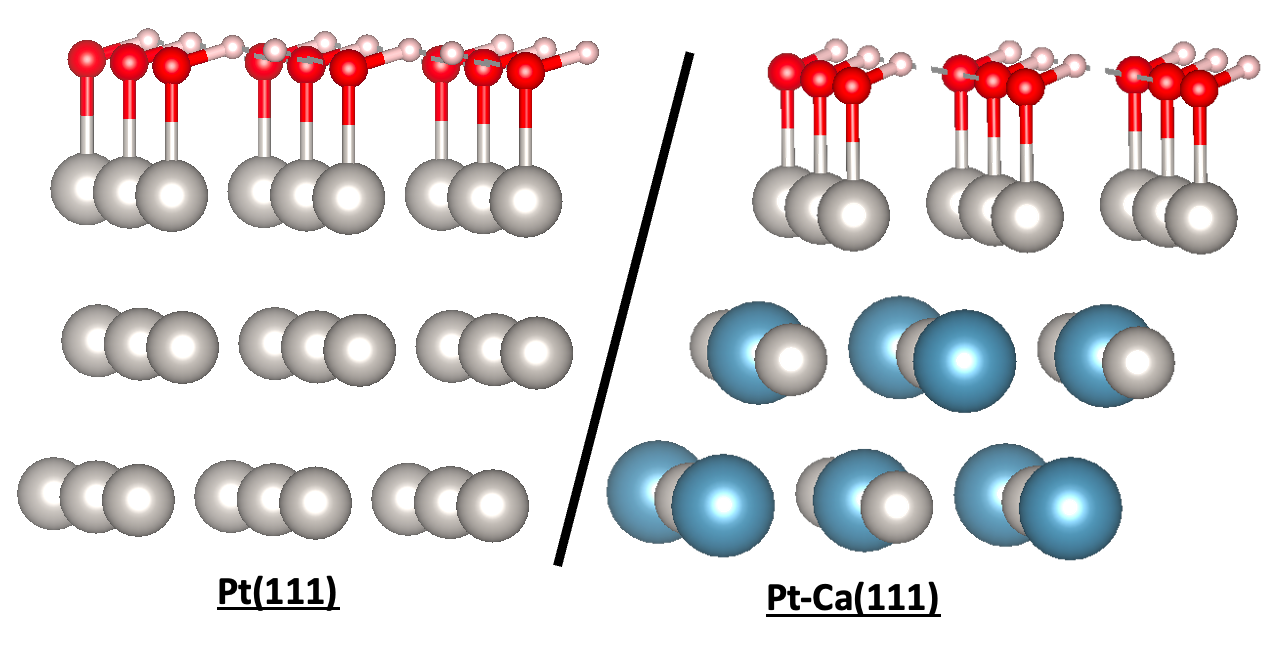


**Figure S29**. Depiction of a stable fully hydroxylated surface (9 *OH, cf. Pourbaix diagram in Figure S30) of Pt(111) and PtCa(111). In the PtCa(111) configuration, Ca is doped in the subsurface layers of the slab, which resembles the core-shell concept, with Pt serving as the active site for ORR.

## **Note 1: Pt-Ca system**

Calcium doping is implemented in the subsurface layers, with Pt serving as the active site at the top layer. This doping approach mimics the concept of the experimentally investigated core-shell nanoparticles. To achieve the Ca-stacked configuration, every alternate Pt atom in the lower layer is substituted with Ca, as depicted in **Figure S29**. The metallic atomic radii of Pt and Ca are approximately 138.5 pm and 197 pm, respectively. This substantial size difference between the two atoms is expected to induce structural reconstruction in the doped layers, thus likely influencing the catalytic properties of the Pt surface atoms, which we discuss in the main text of our work.

## **Note 2: Modeling of electrochemical processes**

To describe the energetics of proton-coupled electron transfer steps relevant to the ORR, we make use of the computational hydrogen electrode (CHE) approach developed by Nørskov and coworkers.^[23]^ The CHE framework based on a canonical (constant charge) formalism significantly simplifies the treatment of electrochemical environments in computational studies. It enables the calculation of free energies for adsorbate configurations at the potential of zero charge, while the influence of the applied electrode potential and pH is incorporated retrospectively. This is achieved by leveraging the electrochemical equilibrium between H^+^/e^-^ and ½H_2_, as illustrated in equation (eq4):

$CHE: H^{+}+e^{-} \rightleftharpoons\frac{1}{2}H_{2} @U=0 vs \mathrm{RHE}, p=1 \mathrm{bar}, T=298.15 K$ (eq4)

To this end, it is sufficient to calculate the free energy of a hydrogen molecule to mimic a proton-electron pair. Besides H_2(g)_, we use H_2_O_(g)_ at *p* = 0.035 bar and *T* = 298.15 K as a second reservoir because water vapor is in equilibrium with liquid water under the specified conditions.^[23]^

It is important to note that the reversible hydrogen electrode (RHE) serves as a typical reference electrode in electrochemical experiments, particularly for electrocatalytic reactions involving equal numbers of proton and electron transfers. Considering that an equal number of proton and electron transfers is met for all intermediate states in the ORR (*vide infra*), we apply the RHE scale as a reference in our computational work.

The ORR is assumed to proceed via different reaction intermediates,^[24]^ including the *OO, *OOH, *O, and *OH adsorbates. To gain insight into the energetics for the formation of these intermediate species on the surface of the Pt and Pt-Ca model systems investigated here, we determine free-energy changes according to equations (eq5):

$\Delta G= {\Delta E}_{tot}-{\Delta E}_{ZPE}-T\Delta S$ (eq5)

Here, ${\Delta E}_{tot}$ represents the adsorption energy based on the electronic energy, which is calculated using DFT. The term ${\Delta E}_{ZPE}$ corresponds to the zero-point energy correction, which is also obtained from DFT calculations. The entropy correction – $T\Delta S$– is considered to account for the vibrational contributions of the surface species as well as translational, vibrational, and rotational contributions from all reference molecules.^[25]^ We refer to our previous work^[26,27]^ for further details related to the determination of the $T\Delta S$term and the determination of adsorption free energies using H_2(g)_ and H_2_O_(g)_ as reference molecules in the analysis.


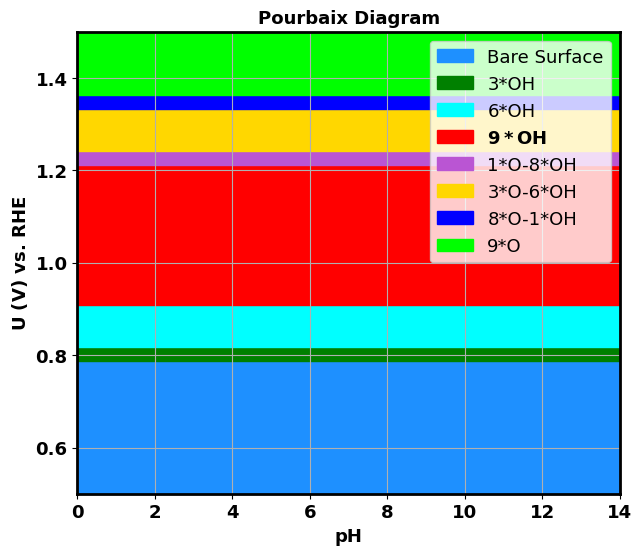


**Figure S30**. Pourbaix diagram for Pt(111) obtained from electronic structure theory calculations in the DFT framework.^[27]^ A fully hydroxylated surface – 9 *OH – is observed under typical ORR conditions (U = 0.93 V vs. RHE).

## **Note 3: Construction of surface Pourbaix diagrams**

To gain insight into the surface structure of the Pt and Pt-Ca systems investigated, we construct a surface Pourbaix diagram to identify stable surface coverages as a function of the applied electrode potential and pH^[28,29]^ During electrocatalytic processes, the interaction of the surrounding aqueous electrolyte with the electrode surface under applied bias leads to the adsorption of gaseous oxygen or water on the electrode surface, thereby forming different surface configurations. It is generally known that the stability of these surface configurations is significantly affected by the applied electrode potential. In a previous work on Pt-based electrodes under ORR conditions, we demonstrated that the Pt(111) surface is mainly occupied by *OH adsorbates^[27]^ (cf. **Figure S30**), forming a fully hydroxylated surface – 9 *OH – under ORR conditions (*U* = 0.93 V vs. RHE). To this end, we adopt the thermodynamically stable
9 *OH surface configuration as the starting point for the investigation of mechanistic pathways in the ORR over Pt and Pt-Ca. For a discussion of the Pourbaix approach, we refer to the literature.^[26,27]^

## **Note 4: Modeling of ORR mechanisms**

The oxygen reduction reaction (ORR) represents a critical performance-limiting step in fuel cells, where hydrogen and oxygen gases react to produce electricity.^[30]^ In this process, four proton-electron pairs are transferred to gaseous oxygen under the formation of two water molecules, as shown in equation (eq6). The inherently sluggish kinetics of the ORR is commonly referred to scaling relations between the intermediate states in the reaction mechanism.^[31]^

$O_{2}+4H^{+}+4e^{-}\to{2H}_{2}O, U^{0}=1.23V vs RHE$ (eq6)

Henceforth, we introduce the most commonly discussed mechanistic pathways in the ORR, thereby following previous work on the topic.^[24]^

***Mononuclear mechanism:***

Several theoretical pathways have been proposed to elucidate the elementary steps of the ORR.^[32]^ Nørskov and co-workers first introduced the mononuclear mechanism,^[23]^ which involves three key intermediates (*OOH, *O, and *OH) at the active site of the electrocatalyst. The elementary reaction steps are summarized below:

| * + O_2(g)_ + (H^+^ + e^-^) ➔ *OOH | Δ*G*_1_ | (eq7) |
| --- | --- | --- |
| *OOH + (H^+^ + e^-^) ➔ *O + H_2_O | Δ*G*_2_ | (eq8) |
| *O + (H^+^ + e^-^) ➔ *OH | Δ*G*_3_ | (eq9) |
| *OH + (H^+^ + e^-^) ➔ * + H_2_O | Δ*G*_4_ | (eq10) |

Here, * denotes an active surface site. In our case, * refers to a Pt atom on the catalyst surface in both the Pt and Pt-Ca systems.

***OOH dissociation (Chem) mechanism:***

Jacob and coworkers outlined the possibility that the *OOH intermediate may dissociate via a chemical pathway, resulting in *O and *OH formation.^[33]^ This chemical dissociation mechanism is described by the following reaction equations:

| * + * + O_2(g)_ + (H^+^ + e^-^) ➔ *OOH+ * | Δ*G*_5_ | (eq11) |
| --- | --- | --- |
| *OOH + * ➔ *O + *OH | Δ*G*_6_ | (eq12) |
| *O + *OH + (H^+^ + e^-^) ➔ *OH + *OH | Δ*G*_7_ | (eq13) |
| *OH + *OH + (H^+^ + e^-^) ➔ *OH + * + H_2_O | Δ*G*_8_ | (eq14) |
| *OH + * + (H^+^ + e^-^) ➔ * + * + H_2_O | Δ*G*_9_ | (eq15) |

***OOH dissociation (EC) mechanism:***

An alternative electrochemical dissociation route for the *OOH intermediate has also been suggested in former works by Jacob and coworkers.^[33]^ The corresponding electrochemical pathway proceeds via the following steps:

| * + * + O_2(g)_ + (H^+^ + e^-^) ➔ *OOH + * | Δ*G*_10_ | (eq16) |
| --- | --- | --- |
| *OOH + * + (H^+^ + e^-^) ➔ *OH + *OH | Δ*G*_11_ | (eq17) |
| *OH + *OH + (H^+^ + e^-^) ➔ *OH + * + H_2_O | Δ*G*_12_ | (eq18) |
| *OH + * + (H^+^ + e^-^) ➔ * + * + H_2_O | Δ*G*_13_ | (eq19) |

***Dissociative mechanism:***

In addition to the *OOH-mediated pathway, oxygen can dissociatively adsorb on the catalyst surface, thereby forming two adjacent *O intermediates.^[33]^ The elementary steps are as follows:

| * + * + O_2(g)_ ➔ *O + *O | Δ*G*_14_ | (eq20) |
| --- | --- | --- |
| *O + *OH + (H^+^ + e^-^) ➔ *OH + *O | Δ*G*_15_ | (eq21) |
| *OH + *O + (H^+^ + e^-^) ➔ *OH + *OH | Δ*G*_16_ | (eq22) |
| *OH + *OH + (H^+^ + e^-^) ➔ *OH + * + H_2_O | Δ*G*_17_ | (eq23) |
| *OH + * + (H^+^ + e^-^) ➔ * + * + H_2_O | Δ*G*_18_ | (eq24) |

***Oxide mechanism:***

In scenarios where adjacent surface sites are occupied by oxide species, an alternative dissociative pathway involves the splitting of the oxygen molecule on *O adsorbates. The oxide-assisted mechanism is represented by the following set of equations^[34]^:

| *O + *O + O_2(g)_ ➔ *OO+ *OO | Δ*G*_19_ | (eq25) |
| --- | --- | --- |
| *OO + *OO + (H^+^ + e^-^) ➔ *OOH + *OO | Δ*G*_20_ | (eq26) |
| *OOH + *OO + (H^+^ + e^-^) ➔ *OOH + *OOH | Δ*G*_21_ | (eq27) |
| *OOH + *OOH + (H^+^ + e^-^) ➔ *OOH + *O + H_2_O | Δ*G*_22_ | (eq28) |
| *OOH + *O + (H^+^ + e^-^) ➔ *O + *O + H_2_O | Δ*G*_23_ | (eq29) |

In order to determine the energetically favored mechanistic description for both the Pt and Pt-Ca systems, we calculate the free-energy changes ${\Delta G}_{j}$ (j = 1, …, 23) and construct free-energy diagrams along the reaction coordinate. The free-energy diagrams are analyzed at *U* = 0.93 V vs. RHE, which corresponds to an applied overpotential value of 300 mV, using a descriptor-based analysis. We apply the activity measure *G*_max_(*U*),^[35,36]^ which is a representation of the energetic span model for electrocatalytic processes at electrified interfaces. For a definition of this descriptor and its application to the ORR, we refer to the literature.^[24,27,37]^


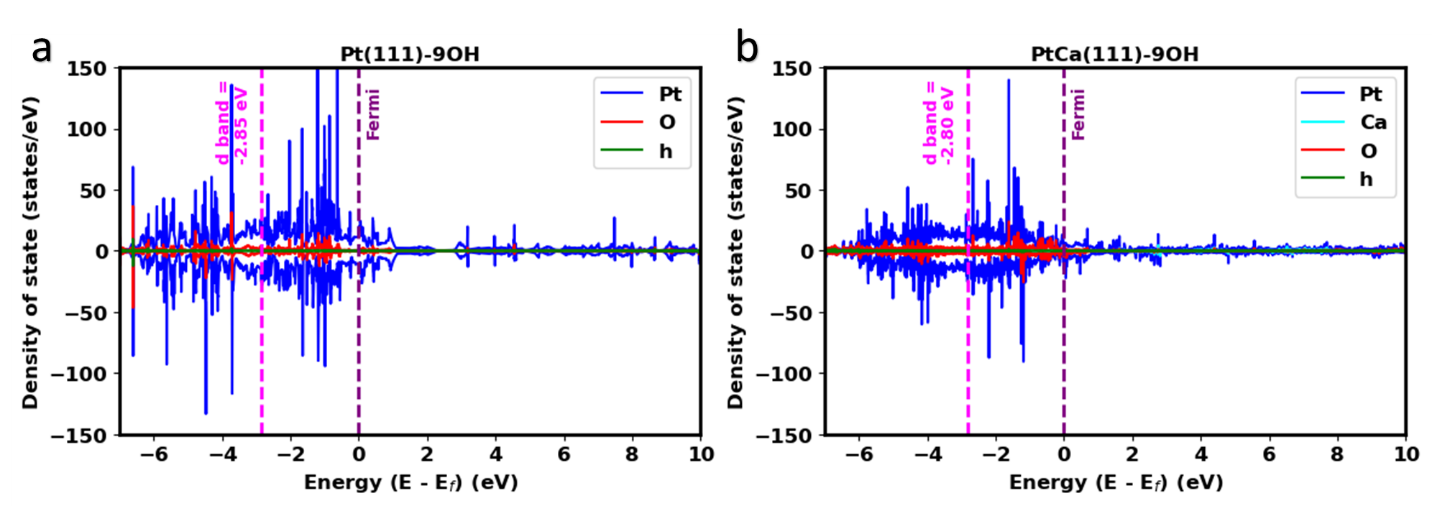


**Figure S31.** Projected density of states (PDOS) for a) Pt(111)-9OH surface, with a d-band center energy of –2.85 eV, and b) PtCa(111)-9OH surface, with a d-band center energy of –2.80 eV.

## **Notes 5: Density of states:**

To comprehend the differences in the electronic structure of the Pt and PtCa model systems investigated, we compute the projected density of states (PDOS) for both systems to determine the d-band center. While our results are discussed in the main text for the pristine surfaces of Pt(111) and PtCa(111), indicating a right shift of the d-band center for PtCa compared to Pt, we demonstrate in **Figure S31** that the right shift of the d-band center also holds if adsorbate coverage under ORR conditions is considered in our calculations.

## **Note 6: Modeling of catalyst stability**

To evaluate the stability of the Pt and PtCa model systems under applied bias, we apply a Born-Haber cycle approach, similar to a recent study by Pacchioni and coworkers.^[38]^ Under anodic reaction conditions, it is possible that a Pt surface atom is leached into the electrolyte to form a divalent Pt ion:

${Pt}_{27}-9OH+H^{+}+e^{-}➔ {Pt}_{26}-8OH+ H_{2}O+ {Pt}_{\left( aq \right)}^{2+}+ {2e}^{-}, G_{overall}$ (eq30)

In this equation, ${Pt}_{27}-9OH$ and ${Pt}_{26}-8OH$ indicates the fully hydroxylated surface and the corresponding surface with a single Pt vacancy, respectively. To calculate the equilibrium potential of the above process, we disentangle equation (eq30) into the following subprocesses using the concept of a Born-Haber cycle:^[38]^

${Pt}_{27}-9OH+H^{+}+e^{-}➔{Pt}_{26}-8OH+ H_{2}O+ Pt(g), G$ (eq31)

$Pt\left( g \right) ➔ Pt(s), G$ (eq32)

$Pt\left( s \right) ➔ {Pt}_{\left( aq \right)}^{2+}+ {2e}^{-}, G$ (eq33)

Note that the free-energy change $G$ can be calculated by DFT calculations, while the free-energy changes $G$ and $G$are taken from thermodynamic data tables.

The overall Gibbs free-energy change for the entire process is given by:

$G_{overall}= G+ G+ G-1eU$ (eq34)

Hence, the equilibrium potential can be calculated according to equation (eq35):

$U_{eq}= \frac{G+ G+ G}{1e}$ (eq35)

We note that the same approach is used for the Pt-Ca case as well. The equilibrium potentials of Pt and PtCa are compared to assess the relative stability of the Pt and PtCa systems under ORR conditions (cf. **Table S1**)

**Table S1.** Free energies for the Born-Haber Cycle of equations (eq30-35)

|  | $\boldsymbol{G}$(eV) | $\boldsymbol{G}$(eV) | $\boldsymbol{G}$(eV) | U_eq_(V) |
| --- | --- | --- | --- | --- |
| Pt(111)-9OH | 4.48 | -5.84^[39]^ | 2*1.188^[40]^ | 1.01 |
| PtCa(111)-9OH | 4.69 |  |  | 1.23 |

**Table S2.** Elemental Composition determined from EDS and XPS.

|  | **EDS** | **XPS** | **ICP-OES** |
| --- | --- | --- | --- |
| PtCa-165 (Pt : Ca) | 27.10 **:** 72.90 | 11.60 **:** 88.40 | 24.98 : 75.02 |
| PtCa-850 (Pt : Ca) | 65.80 **:** 32.20 | 57.20 **:** 42.80 | 60.84 : 39.16 |
| PtCa-After-ADT (Pt : Ca) | 77.80 **:** 22.20 | 70.55 : 29.45 | 80.62 : 19.38 |

## **Table S3.** Structural information and fitting parameters obtained from Pt L_3_-edge EXAFS spectra of Pt Foil, PtCa/C, and PtO_2_. (S0^2^ = 0.8426).

| **Name** | **Shell** | **N** | **R(Å)** | **σ^2^(Å^2^)** | **ΔE_0_ (eV)** | | **R Factor** |
| --- | --- | --- | --- | --- | --- | --- | --- |
| Pt-Foil | Pt-Pt (I) | 12 | 2.760683 ± 0.002621 | 0.00480 ± 0.00030 | 8.59 ± 0.060 | | 0.01282 |
|  | Pt-Pt (II) | 6 | 3.90700 ± 0.0159820 | 0.00802 ± 0.00090 |  |  |  |
| Pt-O_2_ | Pt-O | 6 | 2.02338 ± 0.010000 | 0.00192 ± 0.00140 | 10.79 ± 0.008 | | 0.01966 |
|  | Pt-Pt (I) | 3.30 ± 0.45 | 3.10513 ± 0.009996 | 0.00375 ± 0.00130 |  |  |  |
| PtCa/C | Pt-Pt (I) | 3.00 | 2.60628 ± 0.06082 | 0.00415 ± 0.02463 | 5.61 ± 0.028 | 0.02305 | |
|  | Pt-Pt (Ia) | 2.41 | 2.69903 ± 0.03367 | 0.01813 ± 0.00210 |  |  |  |
|  | Pt-Ca | 2.31 | 3.28869±0.1229 | 0.00000 ± 0.03040 |  |  |  |

## **Table S4.** ORR Half-Cell and Full-Cell Mass activity and durability of reported Pt-alkaline earth, Pt-lanthanide, Pt-early transition alloys.

| **Number** | **Catalyst** | [**MA or jK values @0.9V**](mailto:MA@0.9V) **in HCl0_4_** | **Loss in initial catalyst activity (%)** | **M.A @ 0.9V PEMFC (A/mg_Pt_)** | **Loss in initial catalyst activity (%)** | **Durability Test Protocol** | **Ref.** |
| --- | --- | --- | --- | --- | --- | --- | --- |
| 1. | Pt_5_Ca | N/A | 43% | N/A | N/A | 10k cycles (Half-Cell) | ^[41]^ |
| 2. | Pt_5_Sr | N/A | 49% | N/A | N/A | 10k cycles (Half-Cell) | ^[41]^ |
| 3. | PtxMg | MA = 0.45 A/mg_Pt_ | N/A | 0.15 | 12.8% | 30k cycles (Full-Cell) | ^[1]^ |
| 4. | Pt_3_Y | j_K_ = 6.8 mA/cm^2^ | NA | N/A | N/A | N/A | ^[42]^ |
| 5. | Pt_3_Sc | j_K_ = 1 mA/cm^2^ | NA | N/A | N/A | N/A | ^[42]^ |
| 6. | Pt_5_Gd | j_K_ = 10.4 mA/cm^2^ | 14% | N/A | N/A | 10k cycles (Half-Cell) | ^[43]^ |
| 7. | Pt_70_Y_30_ | j_K_ =28.2 mA/cm^2^ | 0% | N/A | N/A | 3k cycles (Half-Cell) | ^[44]^ |
| 8. | Pt_5_La | jK = 6.8 mA/cm^2^ | 8% | N/A | N/A | 10k cycles (Half-Cell) | ^[45]^ |
| 9. | PtxY | MA =3.05 A/mg_Pt_ | 37% | N/A | N/A | 9k cycles (Half-Cell) | ^[46]^ |
| 10 | Pt_5_Ce | jK =7.4 mA/cm^2^ | 10% | N/A | N/A | 10k cycles (Half-Cell) | ^[47]^ |
| **11.** | **Pt/C** | **MA =0.31 A/mg_Pt_** | **51%** | **0.25** | **55%** | **30k cycles**  **(Full-Cell)** | **This work** |
| **12.** | **PtCa/C** | **MA =0.80 A/mg_Pt_** | **12%** | **0.45** | **19%** | **30 k cycles**  **(Full Cell)** | **This work** |

**References**

[1] E. B. Tetteh, H.-Y. Lee, C.-H. Shin, S. Kim, H. C. Ham, T.-N. Tran, J.-H. Jang, S. J. Yoo, J.-S. Yu, *ACS Energy Lett.* **2020**, *5*, 1601–1609.

[2] E. S. Jeong, I. H. Hwang, S. W. Han, *Curr. Appl. Phys.* **2024**, *59*, 25–32.

[3] *US Department of Energy, Fuel Cells*, **2016**.

[4] P. Hohenberg, W. Kohn, *Phys. Rev.* **1964**, *136*,

[5] W. Kohn, L. J. Sham, *Phys. Rev.* **1965**, *140*,

[6] G. Kresse, J. Furthmüller, *Phys. Rev. B - Condens. Matter Mater. Phys.* **1996**, *54*, 11169–11186.

[7] D. Joubert, *Phys. Rev. B - Condens. Matter Mater. Phys.* **1999**, *59*, 1758–1775.

[8] G. Kresse, J. Furthmüller, *Comput. Mater. Sci.* **1996**, *6*, 15–50.

[9] J. P. Perdew, K. Burke, M. Ernzerhof, **1996**.

[10] G. Kresse, J. Hafner, *Phys. Rev. B* **1993**, *48*, 13115–13118.

[11] K. Mathew, R. Sundararaman, K. Letchworth-Weaver, T. A. Arias, R. G. Hennig, *J. Chem. Phys.* **2014**, *140*, 84106.

[12] E. Caldeweyher, C. Bannwarth, S. Grimme, *J. Chem. Phys.* **2017**, *147*, 34112.

[13] E. Caldeweyher, S. Ehlert, A. Hansen, H. Neugebauer, S. Spicher, C. Bannwarth, S. Grimme, *J. Chem. Phys.* **2019**, *150*, 154122.

[14] T. Y. Yoo, J. Lee, S. Kim, M. Her, S. Y. Kim, Y. H. Lee, H. Shin, H. Jeong, A. K. Sinha, S. P. Cho, Y. E. Sung, T. Hyeon, *Energy Environ. Sci.* **2023**, *16*, 1146–1154.

[15] L. Chong, J. Wen, J. Kubal, F. G. Sen, J. Zou, J. Greeley, M. Chan, H. Barkholtz, W. Ding, D.-J. Liu, *Science* **2018**, *362*, 1276–1281.

[16] X. Tian, X. Zhao, Y. Q. Su, L. Wang, H. Wang, D. Dang, B. Chi, H. Liu, E. J. M. Hensen, X. W. Lou, B. Y. Xia, *Science.* **2019**, *366*, 850–856.

[17] J. Li, S. Sharma, X. Liu, Y. T. Pan, J. S. Spendelow, M. Chi, Y. Jia, P. Zhang, D. A. Cullen, Z. Xi, H. Lin, Z. Yin, B. Shen, M. Muzzio, C. Yu, Y. S. Kim, A. A. Peterson, K. L. More, H. Zhu, S. Sun, *Joule* **2019**, *3*, 124–135.

[18] J. Liang, N. Li, Z. Zhao, L. Ma, X. Wang, S. Li, X. Liu, T. Wang, Y. Du, G. Lu, J. Han, Y. Huang, D. Su, Q. Li, *Angew. Chemie Int. Ed.* **2019**, *58*, 15471–15477.

[19] Z. Qiao, C. Wang, C. Li, Y. Zeng, S. Hwang, B. Li, S. Karakalos, J. Park, A. J. Kropf, E. C. Wegener, Q. Gong, H. Xu, G. Wang, D. J. Myers, J. Xie, J. S. Spendelow, G. Wu, *Energy Environ. Sci.* **2021**, *14*, 4948–4960.

[20] Q. Cheng, S. Yang, C. Fu, L. Zou, Z. Zou, Z. Jiang, J. Zhang, H. Yang, *Energy Environ. Sci.* **2022**, *15*, 278–286.

[21] Z. Zhao, Z. Liu, A. Zhang, X. Yan, W. Xue, B. Peng, H. L. Xin, X. Pan, X. Duan, Y. Huang, *Nat. Nanotechnol. 2022 179* **2022**, *17*, 968–975.

[22] Z. Zhao, M. D. Hossain, C. Xu, Z. Lu, Y. S. Liu, S. H. Hsieh, I. Lee, W. Gao, J. Yang, B. V. Merinov, W. Xue, Z. Liu, J. Zhou, Z. Luo, X. Pan, F. Zaera, J. Guo, X. Duan, W. A. Goddard, Y. Huang, *Matter* **2020**, *3*, 1774–1790.

[23] J. K. Nørskov, J. Rossmeisl, A. Logadottir, L. Lindqvist, J. R. Kitchin, T. Bligaard, H. Jónsson, *J. Phys. Chem. B* **2004**, *108*, 17886–17892.

[24] K. S. Exner, *ChemCatChem* **2023**, *15*, e202201222.

[25] G. Editor, A. Russell, M. Otani, I. Hamada, O. Sugino, Y. Morikawa, Y. Okamoto, T. Ikeshoji, S. A. Wasileski, M. J. Janik, P. Chem, Y. Shen, M. Träuble, G. Wittstock, Q.-S. Chen, S.-G. Sun, Z.-Y. Zhou, Y.-X. Chen, S.-B. Deng, H. Miyake, T. Okada, G. Samjeské, M. Osawa, P. Strasser, S. Koh, J. Greeley, Y. Pluntke, L. A. Kibler, D. M. Kolb, J. Solla-Gullón, F. J. Vidal-Iglesias, A. López-Cudero, E. Garnier, J. M. Feliu, A. Aldaz, P. Chem Chem, A. R. Kucernak, C. Susut, G. B. Chapman, Y. Tong, H. A. Hansen, J. Rossmeisl, J. K. Nørskov, H. Wang, L. Alden, F. J. DiSalvo, H. Siwek, M. Lukaszewski, A. Czerwinski, V. Del Colle, A. Berná, G. Tremiliosi-Filho, E. Herrero, J. M. Jin, W. F. Lin, P. A. Christensen, J. Fuhrmann, H. Zhao, E. Holzbecher, H. Langmach, M. Chojak, R. Halseid, Z. Jusys, J. Behm, M. Michel, F. Ettingshausen, F. Scheiba, A. Wolz, C. Roth, G. García, M. T. M Koper, H. E. Hoster, A. Bergbreiter, P. M. Erne, T. Hager, H. Rauscher, R. J. Behm, *Phys. Chem. Chem. Phys.* **2008**, *10*, 3722–3730.

[26] M. López, K. S. Exner, F. Viñes, F. Illas, *Adv. Theory Simulations* **2023**, *6*, 2200217.

[27] T. Wu, K. Dhaka, M. Luo, B. Wang, M. Wang, S. Xi, M. Zhang, F. Huang, K. S. Exner, Y. Lum, *Angew. Chemie Int. Ed.* **2024**, e202418691.

[28] T. Fuchs, J. Drnec, F. Calle-Vallejo, N. Stubb, D. J. S. Sandbeck, M. Ruge, S. Cherevko, D. A. Harrington, O. M. Magnussen, *Nat. Catal. 2020 39* **2020**, *3*, 754–761.

[29] A. E. Russell, *Phys. Chem. Chem. Phys.* **2008**, *10*, 3607–3608.

[30] A. Kulkarni, S. Siahrostami, A. Patel, J. K. Nørskov, *Chem. Rev.* **2018**, *118*, 2302–2312.

[31] M. Luo, M. T. M. Koper, *Nat. Catal. 2022 57* **2022**, *5*, 615–623.

[32] L. Zhong, S. Li, *ACS Catal.* **2020**, *10*, 4313–4318.

[33] J. A. Keith, G. Jerkiewicz, T. Jacob, *ChemPhysChem* **2010**, *11*, 2779–2794.

[34] T. Binninger, M. L. Doublet, *Energy Environ. Sci.* **2022**, *15*, 2519–2528.

[35] S. Razzaq, K. S. Exner, *ACS Catal.* **2023**, *13*, 1740–1758.

[36] K. S. Exner, *ACS Catal.* **2020**, *10*, 12607–12617.

[37] M. Usama, S. Razzaq, K. S. Exner, *ACS Phys. Chem. Au* **2024**

[38] G. Di Liberto, L. Giordano, G. Pacchioni, *ACS Catal.* **2024**, *14*, 45–55.

[39] A. M. Halpern, *J. Chem. Educ.* **2012**, *89*, 592–597.

[40] A. J. Bard, L. R. Faulkner, H. S. White, Electrochemical Methods: Fundamentals and Applications

[41] U. G. Vej-Hansen, M. Escudero-Escribano, A. Velázquez-Palenzuela, P. Malacrida, J. Rossmeisl, I. E. L. Stephens, I. Chorkendorff, J. Schiøtz, *Electrocatalysis* **2017**, *8*, 594–604.

[42] J. Greeley, I. E. L. Stephens, A. S. Bondarenko, T. P. Johansson, H. A. Hansen, T. F. Jaramillo, J. Rossmeisl, I. Chorkendorff, J. K. Nørskov, *Nat. Chem.* **2009**, *1*, 552–556.

[43] M. Escudero-Escribano, A. Verdaguer-Casadevall, P. Malacrida, U. Grønbjerg, B. P. Knudsen, A. K. Jepsen, J. Rossmeisl, I. E. L. Stephens, I. Chorkendorff, *J. Am. Chem. Soc.* **2012**, *134*, 16476–16479.

[44] S. Jong Yoo, S. K. Kim, T. Y. Jeon, S. Jun Hwang, J. G. Lee, S. C. Lee, K. S. Lee, Y. H. Cho, Y. E. Sung, T. H. Lim, *Chem. Commun.* **2011**, *47*, 11414–11416.

[45] I. E. L. Stephens, A. S. Bondarenko, U. Grønbjerg, J. Rossmeisl, I. Chorkendorff, *Energy Environ. Sci.* **2012**, *5*, 6744–6762.

[46] P. Hernandez-Fernandez, F. Masini, D. N. Mccarthy, C. E. Strebel, D. Friebel, D. Deiana, P. Malacrida, A. Nierhoff, A. Bodin, A. M. Wise, J. H. Nielsen, T. W. Hansen, A. Nilsson, I. E. L. Stephens, I. Chorkendorff, **2014**,

[47] P. Malacrida, M. Escudero-Escribano, A. Verdaguer-Casadevall, I. E. L. Stephens, I. Chorkendorff, *J. Mater. Chem. A* **2014**, *2*, 4234–4243.
